# Circuit topology analysis of cellular genome reveals signature motifs, conformational heterogeneity, and scaling

**DOI:** 10.1016/j.isci.2022.103866

**Published:** 2022-02-05

**Authors:** Barbara Scalvini, Helmut Schiessel, Anatoly Golovnev, Alireza Mashaghi

**Affiliations:** 1 Medical Systems Biophysics and Bioengineering, Leiden Academic Centre for Drug Research, Faculty of Science, Leiden University, Einsteinweg 55, 2333CC Leiden, the Netherlands; 2 Centre for Interdisciplinary Genome Research, Faculty of Science, Leiden University, Einsteinweg 55, 2333CC Leiden, the Netherlands; 3 Cluster of Excellence Physics of Life, Technical University of Dresden, 01062 Dresden, Germany

**Keywords:** Genomics, In silico biology, Mathematical biosciences

## Abstract

Reciprocal regulation of genome topology and function is a fundamental and enduring puzzle in biology. The wealth of data provided by Hi-C libraries offers the opportunity to unravel this relationship. However, there is a need for a comprehensive theoretical framework in order to extract topological information for genome characterization and comparison. Here, we develop a toolbox for topological analysis based on Circuit Topology, allowing for the quantification of inter- and intracellular genomic heterogeneity, at various levels of fold complexity: pairwise contact arrangement, higher-order contact arrangement, and topological fractal dimension. Single-cell Hi-C data were analyzed and characterized based on topological content, revealing not only a strong multiscale heterogeneity but also highly conserved features such as a characteristic topological length scale and topological signature motifs in the genome. We propose that these motifs inform on the topological state of the nucleus and indicate the presence of active loop extrusion.

## Highlights

• Circuit topology quantifies heterogeneity in genomic arrangement
• Scale analysis reveals a characteristic length scale of 10 Mb in genome topology
• We identify highly conserved topological structures related to loop extrusion
• We suggest a topological model of chromatin arrangement for loop extrusion, the *L-loop*

## Graphical Abstract

**Figure:**
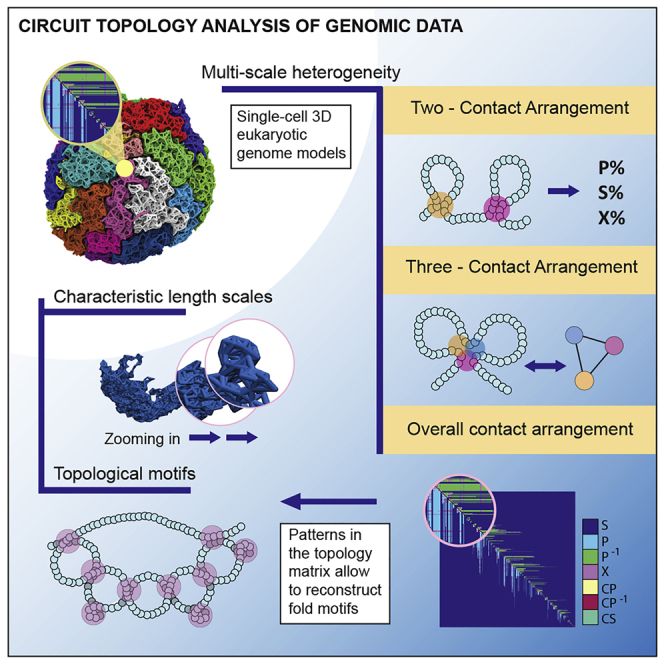

## Introduction

Eukaryotic genomes are efficiently packaged into the nucleus, where they display complex and highly organized 3D structures (Di Pierro, 2019; Gibcus and Dekker, 2013; Rowley and Corces, 2018; Serizay and Ahringer, 2018; Stephens et al., 2019). Small structural variations of this complex architecture can disrupt higher-order chromatin arrangement and modify gene expression and basic regulation (Spielmann et al., 2018). Highly conserved features of genomic organization reveal a complex system characterized by a hierarchical structure of chromatin loops, nested, partially overlapping Topologically Associating Domains (TADs), and compartments (Norton et al., 2018; Rowley and Corces, 2018). Evidence shows that even subtle changes in chromatin looping affect in a meaningful way contact propensity, which in turn is thought to be a fundamental mechanism for the modulation of gene expression (Greenwald et al., 2019). A growing number of studies suggest that alteration in genome topology is related to epigenetic dysfunctions, leading to cancer progression (Flavahan et al., 2019). Detecting these alterations can be a promising diagnostic tool (Hadi et al., 2020). Moreover, a progressively deeper understanding of the complex relationship between topology and gene regulation is opening the way to novel treatments that specifically target genomic 3D organization (Kantidze et al., 2019).

A significant boost in our understanding of genomic structure and its function was provided in recent years by the development of a variety of conformation capture technologies such as Hi-C (Lieberman-Aiden et al., 2009; Pucéat, 2021). These allowed for genome-wide mapping of chromatin interactions for a population of cells, the identification of TADs, and higher-order structures. Moreover, single-cell Hi-C constituted a major advancement in the field, revealing a wealth of highly variable cell-to-cell chromosome conformations (Nagano et al., 2013; Stevens et al., 2017): the highly modular genomic organization coexists with the dynamic and heterogeneous nature of chromosome structure (Nagano et al., 2013, 2017; Stevens et al., 2017). The abundance of available data ensured by Hi-C and single-cell techniques has given rise to significant efforts to provide a suitable framework for topological analysis (Ashoor et al., 2020; Carrière and Rabadán, 2020; Hadi et al., 2020; Norton et al., 2018). For example, graph analysis was used to uncover subgroups of tumors associated with DNA repair defects, which correlate with prognosis severity (Hadi et al., 2020). Persistent homology and network topology have been used to identify recurring patterns at various levels of chromosome structure hierarchy (Carrière and Rabadán, 2020; Norton et al., 2018). However, these frameworks have serious limitations. Both network topology and persistent homology are mostly focused on connectivity, which cannot describe the actual arrangement of the fold and provide a qualitative description of three-dimensional motifs.

**Figure 1:**
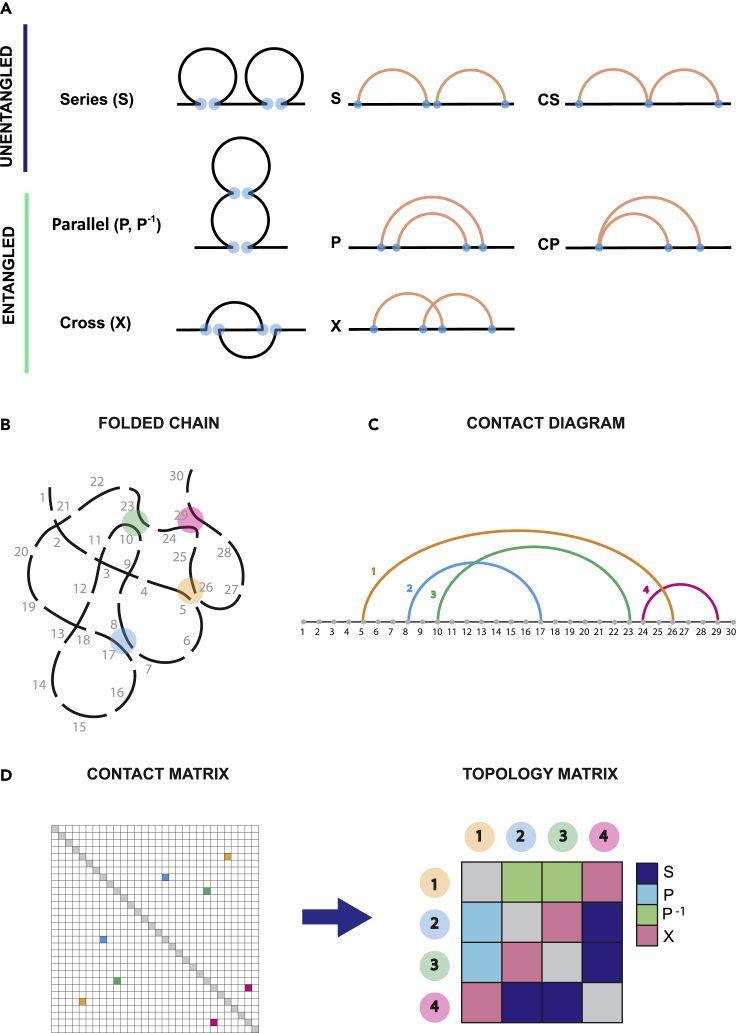
Circuit topology classifies chains based on the pairwise arrangement of intra-chain contacts (A) Circuit topology relations: contacts can be in either one of three topological relations: series, parallel, and cross. Parallel and cross are grouped together as *entangled* relations. (B) Schematic representation of a folded 3D chain. The chain is divided into segments, which in our case can be interpreted as the 100 kb chromosome portions which make up our 3D structures. Contacts can be identified for those segments in the chain that lie closer to each other than the chosen cutoff. Often adjacent segments participate in the same contact (as might be for example the case for segments 26 and 27, which are both close to segment 5). In this case the arrangement gives rise to what we call concerted topological relations (CS, CP). In this scheme, we ignore concerted relations for simplicity. (C) We can represent contacts in the chain as a linear diagram. This representation is useful to visualize the topological relations between contacts. For example, it becomes apparent that contact 1 and contact 4 are in cross relation with each other, as the two respective arcs cross paths. (D) The size of the topology matrix is N x N (where N is the number of contacts). If we compare contact map and topology matrix, we see that each contact in the contact map is represented on both rows and columns of the topology matrix. Elements on the diagonal represent the topological interaction of the contact with itself; although this is defined theoretically as parallel, such relations are excluded from the analysis for lack of physical meaning. Each element of the matrix represents the topological relation of a pair of contacts in the chain. There is a significant reduction in dimensionality, going from a contact map to its topology matrix, which makes the topology matrix easier to handle computationally. In the topology matrix, we lose the spatial information, because we do not know how far the contacts are from each other in the 3D space. However, we gain information about reciprocal contact arrangement.

Our aim is to propose a topological toolbox based on circuit topology (CT) (Golovnev and Mashaghi, 2020; Heidari et al., 2020; Mashaghi et al., 2014; Scalvini et al., 2020; Schullian et al., 2020), capable of detecting not only recurring topological features in genome structure but also of quantifying cell-to-cell variability. CT is the only topology framework for folded linear polymers that categorizes the arrangement of polymer loops or their associated contacts and complements the well-established knot theory (where contacts are typically ignored). Circuit topology analysis of model polymers has provided important insights into complex folding processes (Heidari et al., 2019; Mugler et al., 2014; Scalvini et al., 2021) but the approach has not been used for genome analysis. According to CT, each pair of contacts can be in either one of the three possible topological relations with each other: series (S), parallel (P), and cross (X) (Figure 1A). Series and cross are symmetric relations: if contact A is in series with contact B, contact B is in series with contact A. Parallel on the other hand is not symmetric. Whenever contact A is completely enveloped by contact B, we say that A is in parallel relation with contact B. On the other hand, contact B is now the outer contact. Therefore, we say that contact B is in inverse parallel relation (P^−1^) with contact A. Whenever one of the two contact sites is shared between two contacts, it gives rise to a particular subgroup of topological arrangement, concerted relations: concerted series (CS) and concerted parallel (CP) (Figure 1A). We show that the approach can be further extended to categorize higher-order arrangements, such as topological clusters (three-contact arrangement) and topological fractals (multiple contact arrangement). Moreover, we provide examples on how to relate local topological parameters to genomic structure and correlate it to biologically relevant information, such as gene expression.

We exploit this method for the topological analysis of 3D structures of individual murine stem-cell genomes with a resolution of 100 kb retrieved by single-cell Hi-C data (Stevens et al., 2017). We show how this method not only measures cell-to-cell variability but also identifies statistically distinct subgroups of chromosomes and cells based on their topological properties. We trace the origin of this heterogeneity to loop extrusion activity, suggesting that number and size of chromatin rosettes might be indicative of overall changes in chromosome structure and topology. Importantly, we identify recurring patterns in genomic structures and report evidence of scale invariance in their topological content, with a characteristic length scale of about 10 Mb. Finally, we propose a model for a highly conserved topological motif, which we call an L-loop. We suggest this model represents conserved structural features of extruded loops. The contact frequency that characterizes an individual L-loop correlates with the overall topological properties of the single-cell genome and promises to be highly informative for what concerns gene expression and regulation.

## Results

### First-order intercellular heterogeneity: cells present different percentages of topological entanglement

**Figure 2:**
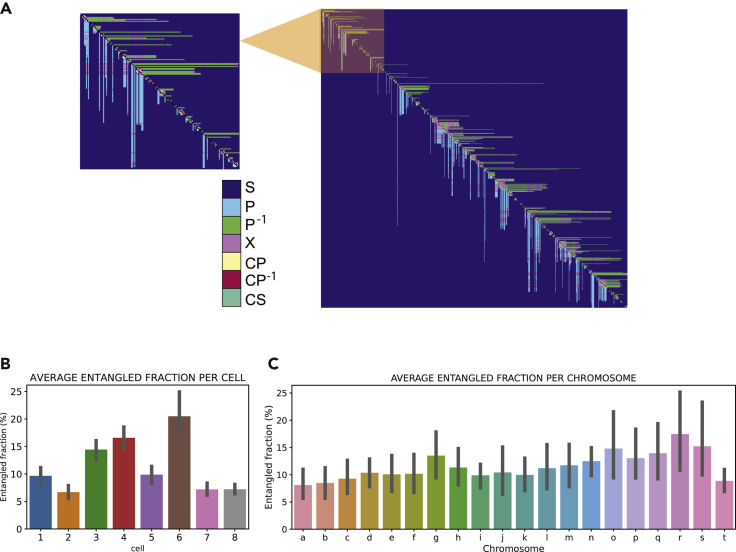
The percentage of entanglement varies from cell to cell (A) Topology matrix of chromosome 1, cell 1 (Stevens et al., 2017). Entangled relations cluster together along the diagonal, indicating a domain-like structure, where domains are in series with each other. (B) Bar plot of the entangled fraction per cell, averaged over all chromosomes. Error bars show a 95% CI for the mean. (C) Bar plot of the entangled fraction per chromosome, averaged over all cells. Error bars show a 95% CI for the mean.

Here we show how CT can be applied to genomic data to reveal cell-to-cell heterogeneity in the topological content. To this end, we apply CT analysis to 3D polymer models retrieved from Hi-C data (Stevens et al., 2017). However, the dependence of the CT framework on contacts alone makes it particularly suitable and easily applicable to Hi-C maps directly. An extensive discussion of opportunities and limitations of this approach is shown in Figure S1. From a conceptual point of view, we can sub-divide CT relations into *entangled* and *unentangled* relations: the former expectedly facilitates local entanglement and globular structures, whereas the latter promotes delocalization of contacts and a linear spatial expansion. It is important to specify that here the word “entangled” is used in a broad sense, as in this paper we are not concerned with knot detection. Once the contacts in the 3D structure have been identified with a spatial cutoff (*r* = 1 particle, 100 kb), the topological relation between each pair of contacts can be stored into an NxN topology matrix, where N is the number of contacts. The topology matrix is fundamentally different from a Hi-C map. Hi-C maps are contact maps, showing the 3D distance between each pair of genomic loci. In the topology matrix, not the genomic loci but contact indexes are displayed along rows and columns: contact 1 (formed by contact sites *i*, *j*), contact 2 (formed by contact sites *k*,*l*), and so on. Contact sites are numbered as they appear along the chain, left end to right end. A detailed scheme on how to go from a contact map to a topology matrix is shown in Figure 1: intrachain contacts are identified and numbered (Figure 1B); from the linear diagram (Figure 1C), it is easy to recognize the three topological relations. This information can then be stored in the topology matrix (Figure 1D), which yields a reduction in dimensionality with respect to the contact map. The topology matrix of a chromosome (Figure 2A) presents a highly ordered distribution of topological relations, with entangled fractions clustering along the diagonal in characteristic elongated features, which we call *L-patterns.* These structures are highly conserved among all chromosomes (Figure S2) and are reminiscent of the plaid-like square patterns found in Hi-C maps (Durand et al., 2016; Lu et al., 2020). However, here we add topological information on top of geometrical information about loci proximity provided by Hi-C; the clustering of entangled relations close to the diagonal not only indicates the tendency of forming tightly packed domains with higher contact density but also informs us on the topological content within these domains. These clusters are highly entangled and assume a looping structure with a highly conserved arrangement. By a first-order analysis, we can characterize a chromosome by the ratio of its entangled fraction over the total of its contacts. Interestingly, the entangled fraction averaged over all chromosomes in a cell varies dramatically from cell to cell (Figure 2B), indicating that the entangled fraction is a good parameter to distinguish nuclei based on their topological properties. We also calculated the characteristic entangled fraction for each chromosome, averaging over all eight cells. Although variations are present (Figure 2C), it appears that intracellular heterogeneity is less dramatic than intercellular heterogeneity (Figure 2B). This suggests that a higher order analysis is needed in order to detect intracellular variations, such as shown below in the local L-loop analysis. Moreover, this indicates that the topology of single chromosomes is most likely widely affected by the overall topological status of the whole genome, which can be quantified in terms of entangled fraction.

### Topological information can be coupled to structural information to gain insight into biological mechanisms

**Figure 3:**
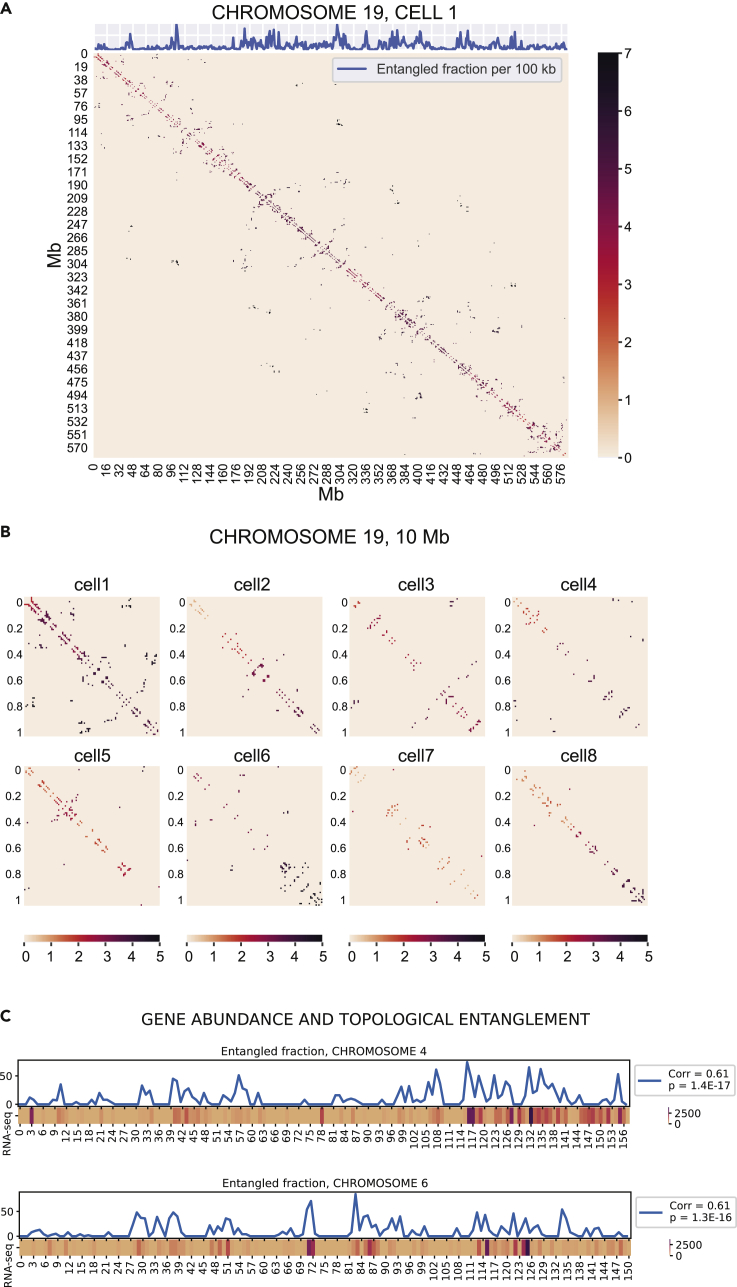
Topological parameters can be projected onto the chromosome sequence and correlated with structural and biological information (A) Contact map of chromosome 19, cell 1, obtained with a spatial cutoff equal to 1.5 particle radii (100 kb). The color scheme represents the number of entangled relationships that each contact entertains with other contacts, as retrieved by the topology matrix. At the same time, we can sum each row of the contact map in (A), to obtain a 1D trace corresponding to the number of entangled relations that each contact site (represented here by a 100 kb chromosomal sequence) experiences. This 1D trace can easily be coupled with other structural and biological information. (B) Zoom over the first 10 Mb of the contact map of chromosome 19, for all cells. The color scheme represents the number of entangled relationships that each contact entertains with other contacts, as retrieved by the topology matrix. (C) Plot of the entanglement fraction trace calculated over population Hi-C maps, coupled with a heatmap of gene abundance data as retrieved by nuclear RNA-seq, for chromosome 4 and 6. The data were coarse-grained in 1 Mb bins. Gene abundance lower than 50 for Mb was set to zero, and a threshold of 250 counts was set over population Hi-C contacts, in order to increase the signal to noise ratio. The label shows the correlation between entangled fraction and gene abundance

The information about entanglement can be traced back to chromosome structure, to gain local topological insight. This insight can then be coupled with biological data, in order to trace a correlation between topological arrangement and chromosomal function. A couple of easy mathematical operations are needed in order to project topological information onto chromosome sequence. As previously mentioned, the topology matrix has size equal to NxN, where N is the number of contacts. Therefore, each row in the matrix corresponds to one specific contact in the structure, and scanning that row, we can access all topological relations that the contact experiences. If we want to know the overall entanglement that the contact experiences, we can sum all entangled relations appearing on that specific row. This information can intuitively be projected onto a contact map, which represents a very common visualization technique for Hi-C data. The result of this procedure is shown in Figure 3A as a heatmap: each pair of contact sites on the two sides of the diagonal is assigned a color depending on the number of entangled relations it experiences in the topology matrix. Fluctuations in the amount of topological entanglement are now visible on the contact maps, allowing us also to compare the pattern thus created among different cells (Figure 3B). For ease of visualization, we zoom on a small portion of chromosome 19, corresponding to 10 Mb (Figure 3B). Once again, we detect a great heterogeneity in the entanglement pattern among the various cells. However, some general features can be identified in most cases, such as a gradual increase in entangled fraction along the first 10 Mb of sequence. Sometimes this increase is not monotonic, as is for example the case in cell 5 and cell 7. Processing these data, we can take a step further and reduce the dimensionality, going from a 2D contact map to a 1D trace of the entangled fraction to be mapped along the chromosome sequence. The procedure is again comparable to what was described for the topology matrix; each row (or column) in the contact map corresponds to all the connections a single contact site experiences, with the addition of a certain weight given by the entanglement value of each contact (represented by the color scheme in the heatmap). Therefore, summing all the values on one row provides a measure of the overall entanglement experienced by each contact site (100 kb of sequence, in this case). This easy matrix operation allows us to map the level of entanglement onto chromosome sequence, as exemplified by the plot on top of the contact map in Figure 3A. Despite its simplicity, this quick procedure yields important practical implications. One could consider to couple this parameter to biological information concerning genetic sequence and look for correlations between entanglement and genetic activity. As a proof of concept of the relevance of this procedure, we tried coupling the entanglement trace with gene abundance data measured by nuclear RNA-seq; the data were provided by Stevens et al. (2017) as supplementary information. In order to provide a good signal-to-noise ratio for this proof of concept, we utilized Hi-C population data; after applying a threshold for excluding contacts with low likelihood (those contacts with counts lower than 250 in the Hi-C map), the CT pipeline thus far described could easily be generalized and implemented for this kind of data. Entanglement data were binned in 1 Mb segments and compared with the RNA-seq data for the corresponding Mb. Figure 3C shows the result of this analysis for chromosomes 4 and 6; the plots for all other chromosomes are displayed in Figure S3. The Pearson correlation coefficient highlights a statistically significant positive correlation between the two parameters, indicating that high entangled fraction might correlate with a higher activity in gene expression. This trend is confirmed for all chromosomes except two, chromosome 10 and 17 (Table 1), which show no correlation. To get a cleaner reading, we set a threshold on gene abundance as well: reads that were lower than 50 per Mb were set to zero. However, overall the correlation coefficients did not change significantly; correlations calculated without threshold for gene abundance are shown in Table S1. Similarly, correlations with different thresholds for Hi-C counts are displayed in Tables S2, S3, and S4 (thresholds set respectively to 100, 200, and 300 counts). The overall trends are not dependent on threshold choices. These results suggest a biological relevance associated with entanglement, as well as confirming the versatility of the method, which can be applied to any biological data from genetic sequence.

**Table 1:** Entangled fraction from Hi-C population map correlates locally with gene expression

Chromosome	Correlation	p value
chr1	0.52	9.4 × 10^−15^
chr2	0.31	2.2 × 10^−5^
chr3	0.46	5.3 × 10^−10^
chr4	0.61	1.4 × 10^−17^
chr5	0.52	9.1 × 10^−12^
chr6	0.61	1.3 × 10^−16^
chr7	0.34	2.5 × 10^−5^
chr8	0.5	2.8 × 10^−9^
chr9	0.22	0.012
chr10	0.03	0.765
chr11	0.53	2.7 × 10^−10^
chr12	0.26	0.003
chr13	0.28	0.002
chr14	0.5	2.6 × 10^−9^
chr15	0.34	0.001
chr16	0.47	1.2 × 10^−6^
chr17	0.07	0.523
chr18	0.53	6.9 × 10^−8^
chr19	0.58	8.8 × 10^−7^
chrX	0.6	9.8 × 10^−18^

Correlation coefficient and p values for entanglement fraction and gene abundance data as measured by nuclear RNA-seq. The data used to obtain these correlation coefficients were coarse-grained into 1 Mb bins. Gene abundance lower than 50 for Mb was set to zero, and a threshold of 250 counts was set over population Hi-C contacts.

### Second-order intercellular heterogeneity: concerted relations and clustering coefficient detect loop extrusion activity

Three-contact topological arrangement appears to be a very powerful tool for genome topology classification, allowing to identify distinct topological states with different physical characteristics. A very handy and well-established mathematical tool for studying higher order topological arrangement is the network clustering coefficient (Kaiser, 2008; Watts and Strogatz, 1998). The clustering coefficient is a measure of the cliquishness of a “neighborhood” in the network: it reflects the extent to which the neighbors of a node (its connections) are also connected with each other. For example, in a social network the clustering coefficient would indicate how many of a person's friends are friends with each other. Let us consider a node n with k_n_ neighbors; the maximum number of edges that can exist among these neighbors is *K*_*n*_ = *k*_*n*_*(k*_*n*_*−1)/2* (Watts and Strogatz, 1998). The local clustering coefficient *C*_*n*_ represents the fraction of these edges that actually exists: *C*_*n*_ = *e*_*n*_/*K*_*n*_, where *e*_*n*_ is the number of existing edges in the neighborhood of n. The clustering coefficient was already applied successfully to Hi-C interaction data in combination with deep learning for the identification of sub-compartments (Ashoor et al., 2020). Here, we exploit the concept of the average clustering coefficient, which means that we consider the average of *C*_*n*_ over all N nodes in the network. We show how the clustering coefficient can detect cellular heterogeneity and exploit CT to explain what is the structural significance of such a parameter.

**Figure 4:**
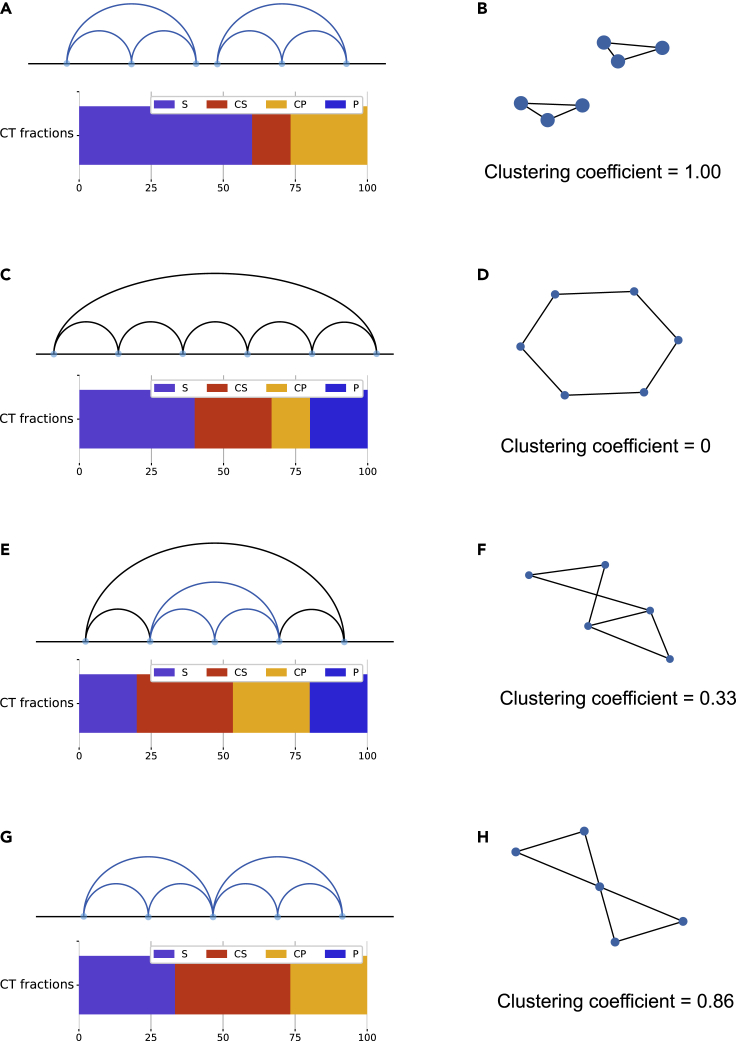
Nonzero clustering coefficient values stem from concerted relations among contacts (A) CT diagram and bar plot with concerted relations, corresponding to clustering coefficient = 1. (B) Network representation of the diagram in (A). (C) CT diagram and bar plot with concerted relations, corresponding to clustering coefficient = 0. (D) Network representation of the diagram in (C). (E) CT diagram and bar plot with concerted relations, corresponding to clustering coefficient = 0.33. (F) Network representation of the diagram in (E). (G) CT diagram and bar plot with concerted relations, corresponding to clustering coefficient = 0.86. (H) Network representation of the diagram in (G).

The interaction network is created by treating contact sites as nodes and contacts as edges. Therefore, coordinates in the polymer that do not participate in any contact are discarded and not considered in the network representation. Now, if we only considered S, P, and X relations, we would obtain a disconnected graph, where edges have only pairwise connections. Such a network would yield a null average clustering coefficient. Therefore, the only topological relations that contribute to the clustering coefficient are concerted relations (Figure 1A), as each node needs to be in contact with at least two other nodes. By looking at the case studies in Figure 4, it becomes apparent that only one possible configuration contributes to the average clustering coefficient of the network, formed by two CS contacts enveloped by a CP contact, which we will call *trefoil* configuration. If the network were composed exclusively by trefoils in series, it would yield a clustering coefficient of 1 (Figures 4A and 4B); on the other hand, if the network were completely devoid of this configuration, the clustering coefficient would be zero (Figures 4C and 4D). The presence of trefoil configurations embedded in structures with other topological relations modulates the clustering coefficient between 0 and 1 (Figures 4E–4H).

**Figure 5:**
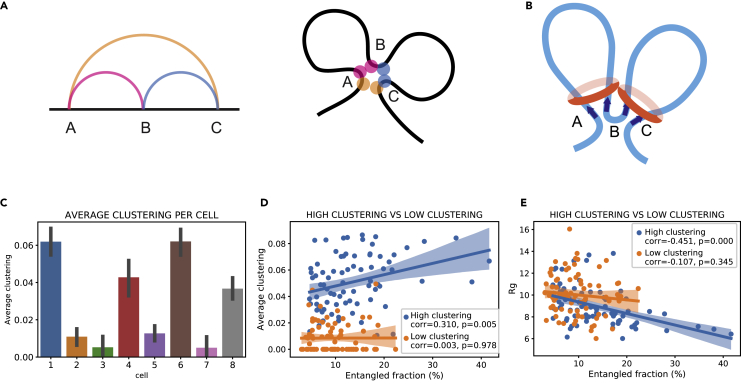
Cells can be divided into topological subgroups depending on their average clustering (A and B) Schematic representation of the type of fold giving rise to the trefoil diagram. (B) Two extrusion complexes bound to DNA during loop extrusion. The complexes stop extruding DNA when they meet on both ends CTCF motifs with convergent orientation. In this case two loops (A-B, B-C) are formed. However, because of the spatial proximity of the loci, this configuration would result in a trefoil configuration in our analysis. (C) Bar plot of the average clustering per cell, averaged over all chromosomes. Error bars show a 95% CI for the mean. (D) Scatterplot of average clustering versus entangled fraction. The high clustering subgroup shows statistically significant positive correlation between the two variables. Dots represent chromosomes from all cells. (E) Scatterplot of gyration radius (per chromosome) versus entangled fraction. The high clustering subgroup displays statistically significant negative correlation between the two variables. Dots represent chromosomes from all cells (Stevens et al., 2017).

Sanborn et al. (2015) previously detected such “cliques” (trefoils) in networks retrieved from population Hi-C data representing an interphase mammalian genome. By combining experimental and computational assays, they were able to identify cohesin-mediated loop extrusion as the mechanism giving rise to this particular network configuration. A model for loop formation based on extrusion complexes associating to DNA was suggested (Alipour and Marko, 2012; Nasmyth, 2001) and later extended with the assumption that each subunit of the extrusion complex recognizes an appropriately oriented motif on a DNA strand, such as a CTCF motif (Sanborn et al., 2015). Such motifs work as “anchors” for the loops, halting the extrusion process whenever the extrusion complex reaches, on the two ends of the loop, a convergent motif pattern (the motifs point toward each other). The trefoil diagram corresponds to two loops coming together at their ends in the 3D space (Figure 5A). Therefore, this picture would correspond to clique loci colocalizing in the same area in the nucleus, giving rise to chromatin rosettes (Figure 5B). Sanborn et al. hypothesized also the formation of a third loop between convergent CTCF motifs, arising from loci A and C. They argued, however, that most likely two consequent loops (A-B, B-C, Figure 5B) tend to form in some cells, whereas the outer loop (A-C) in others. Because we define contacts based solely on spatial cutoffs, we cannot discriminate between an A-C loop formed by loop extrusion complexes, from one created by accidental spatial proximity. However, a cell where mostly (A-B, B-C) loops tend to form would yield in our analysis very different clustering coefficient values than one where mostly A-C loops form, as the latter would not give rise to trefoil configurations.

In order to estimate the prominence of such structures in single cells, we calculated the average clustering coefficient for every chromosome and averaged it for each cell (Figure 5C); astonishingly, clustering values for each cell divide the population neatly into two subgroups, 4 cells with high and 4 with low clustering (approximatively above and below 0.02). There is room for speculation as to what this sharp division might mean. It is possible that we are indeed observing cells where mostly A-B and B-C loops arise (high clustering), as opposed to cells where A-C loops arise, confirming Sanborn et al.’s (2015) hypothesis concerning the two processes being mutually exclusive. This mutual exclusion might explain why we get such stark sub-division among clustering coefficient values among cells.

These two populations display distinct topological properties. We find a weak but statistically relevant correlation between average clustering and entangled fraction (r = 0.31, p = 0.01) for high clustering cells, whereas there is no correlation for the low clustering group (Figure 5D). Similarly, the gyration radius of chromosomes correlates negatively with entangled fraction for high clustering cells (r = −0.45, p = 0.00), while again, there is no correlation for the low clustering cells (Figure 5E). This result is somewhat surprising, as some degree of correlation between geometrical and topological properties would be expected, being the nucleus a tightly packed constrained space (Kinney et al., 2018; Satarifard et al., 2017). These findings suggest that loop extrusion and the formation of chromatin rosettes might be related to wider changes in the overall genomic physical and geometrical properties.

### Higher-order intercellular heterogeneity: fractal dimension of the topology matrix quantifies the topological state of the chromosome

**Figure 6:**
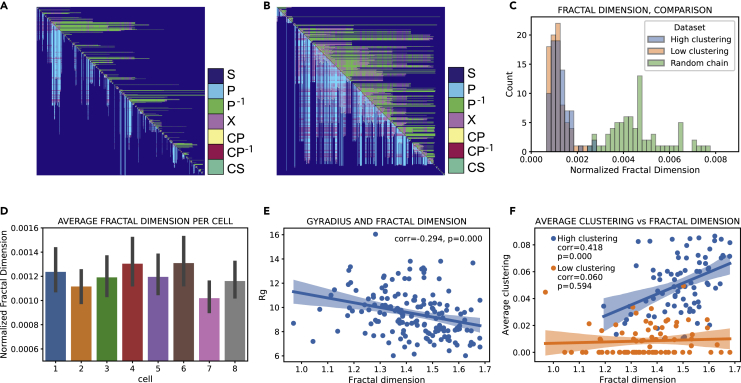
Fractal dimension quantifies the overall contact arrangement in the chromosome (A) Topology matrix of chromosome 5, cell 1 (649 contacts) (Stevens et al., 2017). (B) Topology matrix of a random chain containing 617 contacts. (C) Distribution of chromosome fractal dimension, normalized by number of contacts, for high and low clustering subgroups, and for 80 randomly generated chains. (D) Bar plot of fractal dimension per cell, normalized by number of contacts and averaged over all chromosomes. Error bars show a 95% CI for the mean. (E) Scatterplot of gyration radius versus fractal dimension. Dots represent chromosomes from all cells. The two variables display statistically significant negative correlation. (F) Scatterplot of average clustering versus fractal dimension. Dots represent chromosomes from all cells. The high clustering subgroup shows statistically significant positive correlation between the two variables

Here, we search for fractality in the topological domain and show that the topological fractal dimension *D* can be used as a parameter to quantify the topological distance between chromosomes. Moreover, *D* can highlight the overall topological features of genomes and how these features differ from those of randomly generated folded chains. Unlike the approaches presented in the previous sections, the fractal dimension *D* accounts for both concerted and nonconcerted relations, as well as more complex topological features such as L-patterns. Fractal properties of genome structure have long been suggested; the fractal globule is one of the most used physical models employed to describe genomic 3D arrangement (Iashina and Grigoriev, 2019; Mirny, 2011; Rao et al., 2014). Moreover, the fractal dimension of coding and noncoding sequences along the linear human genome sequence was calculated (Garte, 2004), revealing ranges of lengths where this is constant and remarkably consistent among chromosomes. Here, we take a leap from geometrical properties, over which the fractal dimension is typically calculated, and consider the topological space exclusively. The topology matrix is not dependent on geometrical parameters such as distance between the contacts and is therefore particularly suitable for the identification of recurring structural patterns. We can confront a typical matrix derived from a chromosome (Figure 6A), to one derived from a randomly generated chain (Figure 6B), with comparable number of contacts. The random chain was created by performing a random walk inside a box, where the number of steps (in units of 100 kb particles) corresponds to the typical length and the size of the box to the typical size of a chromosome.

The two matrices look remarkably different, especially in the number and prevalence of entangled fractions, which are confined in ordered structures along the diagonal in the case of chromosomes. This arrangement indicates a more linear development in space of genomic loops and a higher degree of segregation of contacts inside domains. In order to quantify these qualitative differences, the fractal dimension was calculated over the two-dimensional topology matrix in its Minkowski–Bouligand variant (Falconer, 2014). The calculation of the fractal dimension over subsequently bigger portions of the topology matrix revealed a constant fractal dimension (ranging from 1 to 1.7) over a wide range of length scales, revealing that the matrix elements form a self-similar fractal in this range (Figure S4). However, the fractal dimension of the chromosomes was found to correlate positively with the total number of contacts in the chain (r = 0.41, p = 7.1 × 10^−8^); therefore, in order to compare chromosomes with each other, we normalized the fractal dimension by the number of contacts. The dataset was divided into a high clustering and a low clustering group, as identified by the average clustering coefficient. The results are shown in Figure 6C: the two distributions for normalized fractal dimension for high and low clustering cells are not Gaussian, have equal variance, and are statistically distinct (p = 0.023). Low clustering chromosomes (mean: 113 ± 4 × 10^−5^) display a statistically smaller normalized fractal dimension than high clustering ones (mean: 125 ± 5 × 10^−5^). This result again suggests that these two groups of cells might belong to two distinct states from a topological point of view.

Even more stunning are the topological differences between chromosomes and random chains in terms of the normalized fractal dimension (Figure 6C). Ten different random extractions of 80 random chains yielded a much wider distribution of values, indicating that the topology of chromosomes occupies a very narrow range of the space of all possible configurations. The random distribution is significantly different from both chromosome distributions for each extraction (for individual p values see Tables S5 and S6). The displacement of the random chain distribution toward higher values can be explained by the tendency of random chains to form more entangled long range contacts as opposed to chromosomes. In fact, our simulated random walks in a box create equilibrium conformations of polymer globules, which are known to correspond to a collection of overlapping random walks (Emanuel et al., 2009; Lua et al., 2004). According to Flory's theorem, each chain section shows ideal chain statistics up to extensions when it reaches the surface of the globule where it is reflected. This means that for sufficiently large chemical distances between monomers (i.e. distances along the chemical backbone) their contact formation probability is random, especially independent of the chemical distance.

The average normalized fractal dimension is also, as the entangled fraction, a valuable tool to distinguish individual cells (Figure 6D), indicating that each nucleus has its own topological fingerprint. We also observe a weak negative correlation between fractal dimension and gyration radius (r = −0.29, p = 0.00, Figure 6E). Interestingly, a correlation between the average clustering and the fractal dimension can only be observed, once again, for the high clustering group (r = 0.42, p = 0.00, Figure 6F), indicating that, for this distinct topological state, there is an interplay between the abundance in trefoil configurations and the overall topological properties of the chromosome.

### Cumulative CT parameter traces reveal characteristic topological length scales

The trend of entangled fraction and fractal dimension measured over subsequently larger portions of the polymer chain reveals evidence of scale invariance in the topological content. We created windows of increasingly higher number of particles, with an increment of five particles (5, 10, 15, 20, …, n-5, n) over which to calculate topological fractions and the fractal dimension. This procedure is performed starting from the left end of the chain and gradually widening the window of analysis to include the right end. Results for the same analysis carried out by processing the chain in the opposite direction can be found in supplementary information (Figure S5). As stated previously, each particle corresponds to 100 kb. This analysis highlighted some very interesting periodical features.

**Figure 7:**
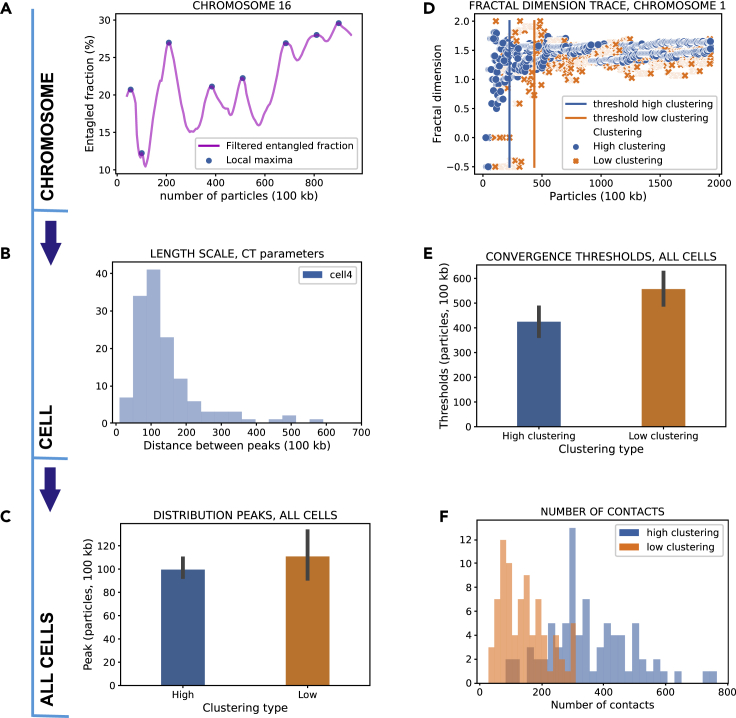
Cumulative CT parameter analysis highlights periodic features and scale invariance (A) Entangled fraction trace of chromosome 16, cell 4 (Stevens et al., 2017). (B) Distribution of distance between local maxima in the chromosome entangled traces, for cell 4. (C) Bar plot of peaks of the distributions of distance between local maxima in entangled traces for all cells. Error bars show a 95% CI for the mean. (D) Trace analysis for fractal dimension, for chromosome 1, all cells. The vertical bars indicate the convergence threshold for the traces. (E) Bar plot of the convergence thresholds of the fractal dimension trace for chromosome 1 in all cells. Error bars show a 95% CI for the mean. (F) Distribution of the number of contacts in each chromosome, all cells. High clustering cells display a significantly higher number of contacts.

By gradually increasing the length scale at which we analyze the chromosome structure, we observe the periodical appearance of local maxima in the entangled fraction (Figure 7A). The tracking of local maxima allowed for the retrieval of the distribution of length scales over which these fluctuations occur (Figure 7B). These distributions are remarkably similar for all chromosomes, with peaks around 10–12 Mb (100–120 particles). There is also no significant difference in the characteristic length scale between the high and low clustering groups (Figure 7C), indicating that this might be a structural feature that is highly conserved among genomic topological states. These conclusions allow for speculations concerning the genomic hierarchical structure, namely the existence of structures with a characteristic length scale of about 10 Mb. What kind of structure in interphase chromosomes is responsible for setting such a scaling? It is possible that this characteristic length might actually still be a "shadow" of the mitotic chromosome state. It has been estimated that the relaxation time of human chromosomes is on the order of 500 years (Rosa and Everaers, 2008), far beyond the lifetime of an organism. This timeline suggests that some features of the mitotic state survive into the interphase state. It has been speculated that both the internal conformations of interphase chromosomes and the fact that each chromosome lives in its own territory can be explained by the fact that the mitotic state of the chromosomes is remembered so that they resemble a dense solution of nonconcatenated polymer rings (Rosa and Everaers, 2008; Sazer and Schiessel, 2018). It seems thus reasonable to speculate that another characteristic feature of mitotic chromosomes (Gibcus et al., 2018), namely their internal helical structure with a repeat length of about 12 Mb, by which the sister chromatids face each other, might still be remembered by the interphase chromosome in the periodic structure of its entangled fraction. This characteristic length scale might be embedded in methods for the detection of structures such as sub-compartments (Ashoor et al., 2020). Moreover, this evidence suggests that between TADs and compartments there might be a more gradual hierarchy of structures, at least from a topological point of view, rather than a sharp structural distinction (Serizay and Ahringer, 2018).

After having defined a characteristic length scale, the question arises whether there is a length scale beyond which genome topology displays scale invariance. An object displaying fractal geometry yields the same fractal dimension regardless of the scale at which the object is observed. Clearly, a chromosome is not a pure geometrical fractal, but an object in the physical world partly displaying fractal properties (Garte, 2004). Therefore, there is a finite interval where the fractal dimension of a chromosome can be considered constant (Figure 7D). We can build a trace of the fractal dimension with the same method used for the entangled fraction and define a threshold after which *D* can be considered constant. By tracking the threshold of a chosen chromosome (chromosome 1) in all eight cells, we find that chromosomes in high clustering cells converge sooner to fractal behavior than those in low clustering cells (Figure 7E), i.e., a smaller number of kb is needed for the chromosome to display fractal properties. These findings should not be surprising if we consider that high clustering cells have a higher contact frequency than low clustering cells (Figure 7F); the distribution of the number of contacts in these two cells is also statistically different (p = 0.0001).

### Recurring motifs in chromatin looping reveal inter- and intracellular heterogeneity

**Figure 8:**
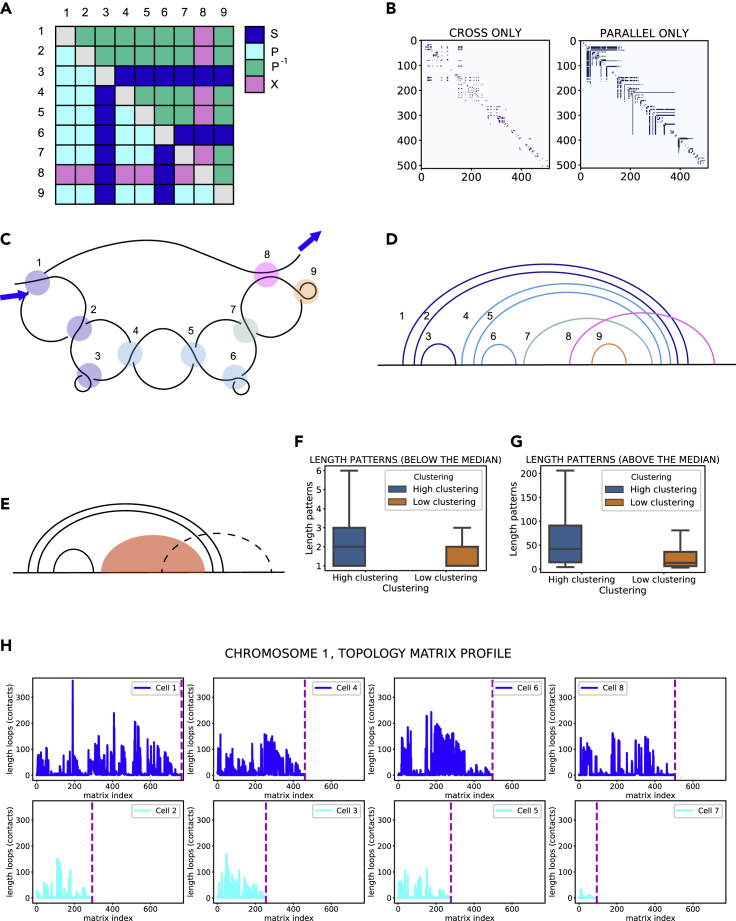
L-loops are topological models for chromatin looping (A) Model matrix of L-patterns. (B) Topology matrix of chromosome X, cell 1 (Stevens et al., 2017), split into its parallel and cross components. (C) Graphical representation of the L-loop corresponding to the matrix in (A). (D) Circuit diagram corresponding to the L-loop in (C). (E) Model circuit diagram of an L-loop. (F and G) Quantile analysis of the length of L-patterns in the matrix. Bar plots of L-pattern lengths for above and below the median of the dataset. Whiskers in the boxplot are extended to 1.5 IQR. (H) Matrix profile of chromosome 1 in all cells. First row: high clustering subgroup. Second row: low clustering subgroup. Dashed lines indicate the end of the topology matrix for each chromosome.

Recurring patterns in the chromosome topology matrix suggest a specific multiscale structure in chromatin arrangement. We call an *L-pattern* a feature of the topology matrix displaying elongated parallel branches interrupted by cross stripes (Figure 8A). These patterns tend to appear as nested in the matrix, often with cross stripes appearing along the same row/column in subsequent patterns (Figure 8B). We suggest a fold model, the L-loop, which can yield such highly conserved patterns in a topology matrix (Figure 8C). The loop originates with one (or more) parallel contact, which appears as a *turn* in the fold (contacts 1 and 2 in Figure 8C). The number of turns defines the thickness of the corresponding L pattern in the matrix. The (one or more) turns are then followed by a smaller contact, which we call a *pocket* (contacts 3,6,9 in Figure 8C). This structure, turn + pocket, can be repeated a few times in a row, as we proceed along the L-loop. In the diagram, these structures appear in parallel with each other (Figure 8D) and ultimately are responsible for the number of nested L-patterns in the matrix. In this case, we have three subsequent turn + pocket motifs, yielding three nested L-patterns in the matrix in 8A. One or more cross contacts may arise, when the loop structure thus created comes into contact with other regions of the DNA strand (contact 8 in Figure 8C). These contacts form the cross stripes perpendicular to the L-patterns in the matrix. The loop develops further after the cross contact, by forming either simple pockets (as is the case of contact 9 in Figure 8C) or new sub-loops. Inspection of the matrices (Figure 8B) shows that often one L-pattern is striped by several cross contacts, indicating that the L-loop comes into contact often with other segments of DNA, effect probably due to the spatial constraints imposed by the nucleus. The total number of contacts enveloped (in parallel relation) in a turn provides the length of the L-pattern in the matrix. For example, turn 1 envelops all other contacts, resulting in a length of 8 in the outer L-pattern. To summarize, Figure 8E displays the basic build of an L-loop: a number of turns followed by a pocket and a cluster of contacts (in red in the Figure), which can contain a variable number of turn + pocket structures, cut through by possible cross contacts (represented with a dashed line). Although the topology matrix presents several small variations of the pattern suggested here (Figure S6), this representation is a useful ideal motif to interpret looping structures in the matrix.

Although these looping structures are present in all chromosomes analyzed in this study, their numbers and lengths vary substantially for both the high and low clustering group. Considering the wide range of sizes that L-loops encompass, it was beneficial to divide them into two quantiles according to their size, above and below the median (Figures 8F–8G). The high clustering group displays a long tail toward higher values, reaching a maximum of 200 contacts for the group above the median. Figure 8H shows the matrix profile, e.g. the size of L loops (number of contacts enveloped by the loop) for chromosome 1, in all eight cells. It is apparent how for the low clustering group not only the total number of L-loops is significantly lower (unsurprising, given the total number of contacts is overall less) but also the contact frequency within the loops is strikingly different.

Considering these results in light of the aforementioned link between clustering coefficient and loop extrusion activity, one might try to draw a relation between the suggested structure for the L-loop and loop extrusion mechanisms. L loops originate by one main contact (the *turn*), which then encompasses all other sub-loops in parallel relation. This mechanism might very well indicate the formation of contact domains by loop extrusion (Fudenberg et al., 2016), implying therefore that the original “turn” is the loop formed by the extrusion complex. Moreover, this structural insight allows us to shed a new light on the positive correlation found earlier in our analysis between local entangled fraction and gene expression. Entangled fraction along rows corresponds to the number of contacts found in an L-loop; it is known that contact propensity participates in a bidirectional correlation with gene expression/regulation (Greenwald et al., 2019). Therefore, it is not surprising that a higher number of contacts in an extruded loop might display correlative patterns with transcriptional activity.

**Figure 9:**
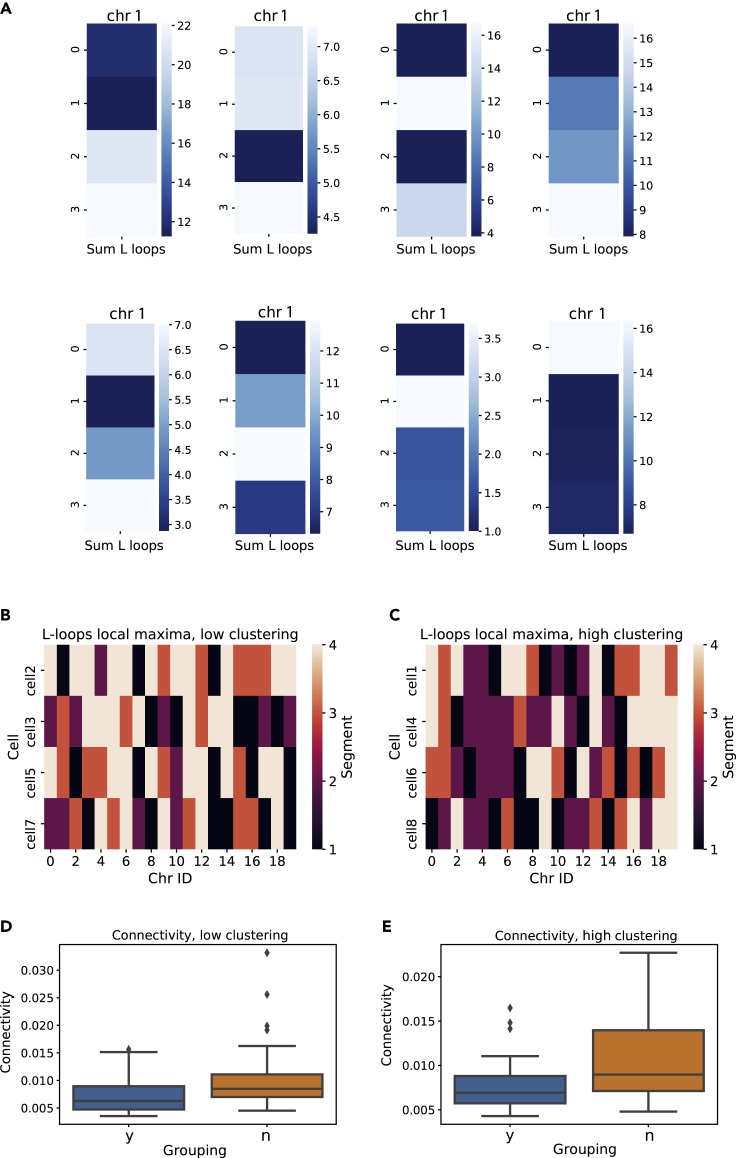
Local distribution of L-loops reveals inter-cellular chromosomal heterogeneity (A) Heatmaps of the sum of contacts enveloped by L-loops, for chromosomes divided into four segments. The figure shows the heatmap for Chromosome 1 in all eight cells (Stevens et al., 2017). (B) Heatmap of the local maxima position of L-loop contacts for each chromosome in low clustering cells. (C) Heatmap of the local maxima position of L-loop contacts for each chromosome in low clustering cells. (D) Boxplot of connectivity for the two groups of chromosomes, based on L-loop contact maxima in Segment 2, for low clustering cells. Whiskers in the boxplot are extended to 1.5 IQR. (E) Boxplot of connectivity for the two groups of chromosomes, based on L-loop contact maxima in Segment 2, for high clustering cells. Whiskers in the boxplot are extended to 1.5 IQR.

The length of L-patterns in the topology matrix can also provide interesting local information, once the chromosome data have been sectioned according to the desired spatial resolution and with an appropriate spatial cutoff. As a proof of concept, we divided the chain into four segments of equal number of beads and computed the total number of contacts contained in L-loops for the section. An example of this analysis carried out with eight segments instead of four is shown in Figure S7. This procedure resulted in chromosome “bar codes” (Figure 9A). It is notable, from the bar code of a single chromosome belonging to different cells, how not only the overall topological properties but also the local arrangement can vary wildly. Interestingly, the bar codes for the total number of contacts clustered in L-loops do not mirror those for the total number of contacts (Figure S8), indicating that there is no direct correspondence between the number of contacts and their arrangements into L-loops. Therefore, the information is far from redundant. The information for all cells and all chromosomes can be conveniently visualized in heatmaps, to identify trends. For example, one might choose to plot where the maximum L-loop contacts appear in each chromosome (Figures 9B and 9C). At a first glance one notices a prevalence of white in the low-clustering cell map (Figure 9B) and of purple in the high clustering one (Figure 9C), indicating that a good portion of chromosomes tend to have their maximum in their last segment when in the low clustering state, whereas they reach it in the second segment when highly clustered. We can ideally divide the chromosomes based on this tendency: Chromosome 1, 2, 3, 5, 8, 10, 11, 18, and 20 reach maxima in segment 2 (purple in the heatmap) in at least one of the cells in the low clustering group, whereas all other chromosomes do not. Therefore, two distinct groups of chromosomes are created based on their local topological arrangement. Quite interestingly, these two groups display different topological properties in both clustering cell groups. Once again, we couple our analysis to a well-known concept in network topology, network connectivity (Figures 9D and 9E). The chromosome group that does not display a maximum length of L-loops in the second segment in the low clustering state has overall higher connectivity, in both cell groups. High connectivity in gene networks was previously shown to be highly informative on disease heritability (Kim et al., 2019). Therefore, local topological arrangements can be a useful predictor about the overall topological properties of the chromosome and potentially about its biological functions as well.

## Discussion

There is growing experimental evidence that the complex and highly organized 3D architecture of genome relates to its function (Cavalli and Misteli, 2013; Flavahan et al., 2019; Greenwald et al., 2019; Ong and Corces, 2014). Various topological frameworks have been tested for chromosome characterization (Ashoor et al., 2020; Carrière and Rabadán, 2020; Hadi et al., 2020; Kim et al., 2019; Norton et al., 2018). Considering the advanced molecular machinery involved into simplification of topological entanglement in DNA (Orlandini et al., 2019; Pouokam et al., 2019), knot theory can hardly provide a universal option for genome characterization. Moreover, knot theory ignores contacts, which are thought to be biologically relevant in health and disease (Flavahan et al., 2019; Greenwald et al., 2019; Hadi et al., 2020; Kantidze et al., 2019). This raises the question of whether contact-based topologies such as CT and network theory can be used to provide an optimal match to the wealth of information provided by technological advances such as Hi-C genome-wide contact libraries (Lu et al., 2020; Nagano et al., 2013; Rao et al., 2014; Stevens et al., 2017). In this article, we demonstrated how CT and network topology can be embedded to provide structural insight into chromosomal organization. We quantified and characterized intercellular heterogeneity at various orders of complexity, from pairwise arrangement of contacts to whole chromosomes; this flexibility of scale is a great advantage considering the highly hierarchical genomic structure (Gibcus and Dekker, 2013). Moreover, the method can easily be adjusted to various levels of data resolution, allowing for a full characterization of genome architecture.

We demonstrated an easy technique to project the topological information retrieved by CT onto contact maps and chromosome sequence (Figure 3). The topological insight thus gained can be correlated with biological information such as gene expression data. Here, we found that the local entanglement fraction correlates positively with nuclear RNA-seq gene abundance data. We also detected two statistically distinct topological states in which we could subdivide the single-cell dataset: high and low clustering states. These states seem to stem from loop extrusion activity. High clustering nuclei present a higher number of chromatin rosettes (Figure 5C), have higher number of contacts clustered in their extruded loops, and have overall higher entangled fraction (Figures 8F–8H). Because we found a correlation between entangled fraction and gene expression, one might speculate that this particular topological state might present a higher transcriptional activity than its low clustering counterpart. Recent studies have suggested that the transcriptional activity of RNA polymerase might be the driver behind the cohesin ring displacement yielding loop extrusion (Brandão et al., 2019; Lengronne et al., 2004). This suggests that transcriptionally active cells show topological states characterized by more chromatin rosettes.

The comparison of the topology matrix of a chromosome with that of a random chain gives insights about chromosome topological composition; the significantly low entangled fraction calls for the classification of chromosomes as *sparsely entangled*. The segregation of entangled contacts into highly conserved patterns allowed for the creation of a model for higher order chromatin looping and loop extrusion, which we called an L-loop. The local density of contacts inside L-loops allowed us to detect not only intercellular but also intracellular heterogeneity, with the detection of subgroupings of chromosomes with statistically different network connectivity. Despite the astonishing multilevel heterogeneity detected, some of the measured features appear to be present in all nuclei. The identification of a characteristic topological length scale in chromosome arrangement is not only significant from a theoretical point of view, but it could also be implemented into algorithms for compartments and subcompartments detection (Ashoor et al., 2020). Therefore, we can conclude that the CT-based topological toolbox offers insight into both chromosome heterogeneity and the universal organizational principles of genomes; as the resolution for single-cell Hi-C data increases, we expect to be able to apply the CT-based technology to both single-cell TAD and sub-TADs territories.

### Limitations of the study

The future applicability of the method strongly relies on its adaptability to Hi-C contact maps, as these constitute the most common data type in the field. The pipeline described in this paper is directly applicable to single-cell contact maps almost completely, with the exception of the “three-contact arrangement” step, which involves the calculation of the average network clustering coefficient. Contact maps store information about contacts in a binary fashion (Figure S1); therefore, they might not carry enough information about contacts formed by triplets of contact sites, such as that depicted in Figure 5A. For this reason, the average clustering coefficient calculated directly over the contact maps (as opposed to the 3D model) of the chromosomes in this paper yielded 0 in all cases. This issue might be resolved by choosing a coarser binning of the contact maps but that would come with a loss in resolution. The issue, however, does not concern contact maps from bulk Hi-C, where the presence of contact counts and likelihoods makes it possible to detect three-contact arrangement and yields therefore a nonzero clustering coefficient. The extension of the pipeline to population Hi-C is possible with the substitution of the spatial cutoff with a cutoff in number of counts. However, a rigorous study on the effect of such a choice in cutoff is still missing and will be the object of future research.

The dataset used in this paper also presents some limitations. The resolution and number of cells could be improved in order to generalize the findings of the study and gain insight into the topological arrangement of sub-TAD structures.

## STAR★Methods

### Key resources table

**Table:** 

REAGENT or RESOURCE	SOURCE	IDENTIFIER
**Deposited data**		

Raw data	Stevens et al., 2017	Expression Omnibus (GEO) repository: GSE80280

**Software and algorithms**		

CT analysis	This paper	Zenodo: https://zenodo.org/badge/latestdoi/428377249
Scipy 1.4.1	Virtanen et al., 2020	https://doi.org/10.1038/s41592-019-0686-2 https://scipy.org/
NetworkX 2.4	Hagberg et al., 2008	https://networkx.org/documentation/networkx-1.10/index.html
Fractal-dimension.py	Nicolas P. Rougier, 2016	https://gist.github.com/rougier/e5eafc276a4e54f516ed5559df4242c0#file-fractal-dimension-py

### Resource availability

#### Lead contact

Further information and requests for resources, data, and codes should be directed to and will be fulfilled by the lead contact, Alireza Mashaghi (a.mashaghi.tabari@lacdr.leidenuniv.nl).

#### Materials availability

This study did not generate new unique reagents.

### Method details

#### Data processing

##### 3D genomic structures

All structures analyzed are previously published bead-on-a-string polymer models recovered from single-cell Hi-C (Stevens et al., 2017), consisting of 20 chromosome structures from eight individual G1-phase haploid mouse embryonic stem (ES) cells. Contacts in the structures were identified by defining a spatial cutoff. The analysis was repeated for cutoffs *r*_*C*_ = 0.5, 1.0, 1.5, 2.0 particle radii (each particle corresponds to 100 kb). All data shown in the paper were obtained by *r*_*C*_ = 1.0 (unless otherwise specified), which was considered to be the ideal cutoff distance by visual inspection of contacts in the chain. However, the general conclusions of our analysis are not dependent on the cutoff used (see Figures S2 and S9). Contacts between first neighbors were excluded. Contact sites were used to build the CT topology matrix (NxN, where N is the number of contacts in the chain) and the networks, by custom made Python code. CT relations (Mashaghi et al., 2014) were assigned based on the mathematical relations summarized below:

$$C_{i,j}SC_{r,s}\Leftrightarrow \left[i,j\right]\cap \left[r,s\right]=\emptyset$$

$$C_{i,j}PC_{r,s}\Leftrightarrow \left[i,j\right]\subset \left(r,s\right)$$

$$C_{i,j}XC_{r,s}\;\Leftrightarrow \left[i,j\right]\cap \left[r,s\right]\notin \left\{\left[i,j\right],\;\left[r,s\right]\right\}\cup \;P\left(\left\{i,j,r,s\right\}\right)$$

$$C_{i,j}CSC_{r,s}\;\Leftrightarrow \left(\left(\left[i,j\right]\cap \left[r,s\right]=\left\{i\right\}\right)\vee \left(\left[i,j\right]\cap \left[r,s\right]=\left\{j\right\}\right)\right)$$

$$C_{i,j}CPC_{r,s}\;\Leftrightarrow \left(\left(\left[i,j\right]\subset \left[r,s\right]\right)\wedge \left(i=r\;\vee j=s\right)\right)$$

P denotes the powerset i.e., all subsets of a set including the null set (⊘). Contact indexes (*i, j, r, s*) were assigned by scanning the chain left end to right end. Networks were built and analyzed by NetworkX (Hagberg et al., 2008), a freely available Python library.

##### Hi-C maps and population data

Topology matrices derived directly from Hi-C maps were constructed by processing the indexes of the contacts, which were used to derive the topological relations. In the case of population Hi-C maps, we considered as coordinates of the contacts the middle point of the genomic loci. When constructing a topology matrix from a Hi-C map, no spatial cutoff was set. Instead, a threshold was set on the number of counts, to select most likely contacts and reduce computational time. The threshold set for the results displayed in the paper is 250, while results for thresholds equal to 100, 200 and 300 are displayed in Tables S2, S3, S4. While projecting the topological information onto the genomic sequence, the entanglement data was binned in 1Mb bins depending on the position along the sequence of one of the two contact sites, the one that happened first along the sequence. The binning of the nuclear RNA-seq gene abundance was binned in a similar way: the abundance of all genes which had middle point in the same 1 Mb bin was summed, to yield the bars in Figure 3C.

#### Random chain generation

The random chains were generated by simulating a random walk on a grid constrained by a box. The step of the grid was set to one particle (100 kb). The total number of steps was set to match the size of each chromosome (80 random chain for 80 chromosomes). The size of the box was assigned starting from the spatial extension of the chromosomes: the maximum extension of chromosomes (in units of particles) on the x, y and z axis defined the length of the box walls. Contacts between first neighbors were excluded, to match the processing procedure of chromosome structures. The chain was not allowed to fold onto itself in two steps (go from position one to position 2, and back to one again in consecutive steps).

### Quantification and statistical analysis

#### Fractal and length scale analysis

The topological fractal dimension was calculated over the two dimensional topology matrix by use of the box counting method (Garte, 2004; Falconer, 2014). Roughly speaking, this method consists in counting the number of boxes required to cover the fractal set on a grid, and observe how this number changes as the grid spacing gets finer. The box-counting fractal dimension of a fractal F is defined as

$$D_{box}\left(F\right)=\lim_{l\to o}\frac{\log\;N\left(l\right)}{\log\left(\frac{1}{l}\right)}$$

Where *N(l)* is the number of boxes of size *l* needed to cover the F set. The code was adapted from a freely available Github repository (Rougier, 2016). A length scale analysis was performed by creating sub-sections of chromosome chains of increasingly bigger size, with an increase of five beads (5, 10, 15, 20, N-5, N); CT parameters were extracted from each sub-section, creating traces as those displayed in Figure 7 for each chromosome. The sectioning procedure was performed from the left end to the right end of the chain. The result of the analysis is independent of the choice of direction (Figure S5). The CT parameter curves were smoothed by a polynomial power filter of the second degree, on a window of 15 beads (1.5 Mb). Local maxima were identified based on peak properties (SciPy (Virtanen et al., 2020): signal.find_peaks), by setting a threshold on peak prominence (0.2 for results displayed in the paper). Results for other choices in prominence are shown in supplementary information (Figure S9), and do not alter the general conclusion of the study.

#### Matrix analysis

Matrices were stored in TIFF format and processed by custom made Jupyter Lab code. Each matrix element was reassigned either 1 or 0 value, whether it contained an entangled or unentangled relation. Then all entangled relations were counted, for each line, starting from the diagonal, thereby yielding the length of each L-pattern in number of contacts.

#### Statistical analysis

All distributions shown and compared in the paper were tested for normality by Shapiro-Wilk test and equal variances by Levene test. Whenever the two distributions resulted to be normal and with equal variances, the Student's T-test was used for comparison. If the two distributions had unequal variance, the Welch's T-test was used instead, while if one or both distributions failed the normality requirement a non-parametric test was preferred (Mann-Whitney U Test in case of equal variance, Kolmogorov-Smirnov otherwise). All tests were two-tailed. All p-values below the 0.05 threshold were considered significant. All correlations were quantified by Pearson correlation coefficient.

## Data Availability

• This paper analyses available published data (Stevens et al., 2017). Accession numbers are listed in the key resources table. • All original codes have been stored in a Github repository and uploaded to Zenodo: https://zenodo.org/badge/latestdoi/428377249. All codes are publicly available at the date of publication. DOIs are listed in the key resources table. • Any additional information required to reanalyze the data reported in this paper is available from the lead contact upon reasonable request.

## References

[bib1] Alipour E., Marko J.F. (2012). Self-organization of domain structures by DNA-loop-extruding enzymes. Nucleic Acids Res..

[bib2] Ashoor H., Chen X., Rosikiewicz W., Wang J., Cheng A., Wang P., Ruan Y., Li S. (2020). Graph embedding and unsupervised learning predict genomic sub-compartments from HiC chromatin interaction data. Nat. Commun..

[bib3] Brandão H.B., Paul P., van den Berg A.A., Rudner D.Z., Wang X., Mirny L.A. (2019). RNA polymerases as moving barriers to condensin loop extrusion. Proc. Natl. Acad. Sci. U S A.

[bib4] Carrière M., Rabadán R., Baas N., Carlsson G., Quick G., Szymik M. (2020). Topological Data Analysis. Abel Symposia.

[bib5] Cavalli G., Misteli T. (2013). Functional implications of genome topology. Nat. Struct. Mol. Biol..

[bib6] Di Pierro M. (2019). Inner workings of gene folding. Proc. Natl. Acad. Sci. U S A.

[bib7] Durand N.C., Robinson J.T., Shamim M.S., Machol I., Mesirov J.P., Lander E.S., Aiden E.L. (2016). Juicebox provides a visualization system for hi-C contact maps with unlimited zoom. Cell Syst..

[bib8] Emanuel M., Radja N.H., Henriksson A., Schiessel H. (2009). The physics behind the larger scale organization of DNA in eukaryotes. Phys. Biol..

[bib9] Flavahan W.A., Drier Y., Johnstone S.E., Hemming M.L., Tarjan D.R., Hegazi E., Shareef S.J., Javed N.M., Raut C.P., Eschle B.K. (2019). Altered chromosomal topology drives oncogenic programs in SDH-deficient GISTs. Nature.

[bib10] Fudenberg G., Imakaev M., Lu C., Goloborodko A., Abdennur N., Mirny L.A. (2016). Formation of chromosomal domains by loop extrusion. Cell Rep..

[bib11] Garte S. (2004). Fractal properties of the human genome. J. Theor. Biol..

[bib12] Gibcus J.H., Dekker J. (2013). The hierarchy of the 3D genome. Mol. Cell.

[bib13] Gibcus J.H., Samejima K., Goloborodko A., Samejima I., Naumova N., Nuebler J., Kanemaki M.T., Xie L., Paulson J.R., Earnshaw W.C. (2018). A pathway for mitotic chromosome formation. Science.

[bib14] Golovnev A., Mashaghi A. (2020). Generalized circuit topology of folded linear chains. iScience.

[bib15] Greenwald W.W., Li H., Benaglio P., Jakubosky D., Matsui H., Schmitt A., Selvaraj S., D’Antonio M., D’Antonio-Chronowska A., Smith E.N., Frazer K.A. (2019). Subtle changes in chromatin loop contact propensity are associated with differential gene regulation and expression. Nat. Commun..

[bib16] Hadi K., Yao X., Behr J.M., Deshpande A., Xanthopoulakis C., Tian H., Kudman S., Rosiene J., Darmofal M., DeRose J. (2020). Distinct classes of complex structural variation uncovered across thousands of cancer genome graphs. Cell.

[bib17] Hagberg A.A., Schult D.A., Swart P.J. (2008). 7th Python in Science Conference (SciPy 2008).

[bib18] Heidari M., Satarifard V., Mashaghi A. (2019). Mapping a single-molecule folding process onto a topological space. Phys. Chem. Chem. Phys..

[bib19] Heidari M., Schiessel H., Mashaghi A. (2020). Circuit topology analysis of polymer folding reactions. ACS Cent. Sci..

[bib20] Iashina E.G., Grigoriev S.V. (2019). Large-scale structure of chromatin : a fractal globule or a logarithmic fractal?. J. Exp. Theor. Phys..

[bib21] Falconer K. (2014).

[bib22] Kaiser M. (2008). Mean clustering coefficients: the role of isolated nodes and leafs on clustering measures for small-world networks. New J. Phys..

[bib23] Kantidze O.L., Luzhin A.V., Nizovtseva E.V., Safina A., Valieva M.E., Golov A.K., Velichko A.K., Lyubitelev A.V., Feofanov A.V., Gurova K.V. (2019). The anti-cancer drugs curaxins target spatial genome organization. Nat. Commun..

[bib24] Kim S.S., Dai C., Hormozdiari F., van de Geijn B., Gazal S., Park Y., O’Connor L., Amariuta T., Loh P.R., Finucane H. (2019). Genes with high network connectivity are enriched for disease heritability. Am. J. Hum. Genet..

[bib25] Kinney N.A., Sharakhov I.V., Onufriev A.V. (2018). Chromosome – nuclear envelope attachments affect interphase chromosome territories and entanglement. Epigenetics Epigenet. Chromatin.

[bib26] Lengronne A., Katou Y., Yokobayashi S., Mori S., Kelly G. (2004). Cohesin relocation from sites of chromosomal loading to places of convergent transcription. Nature.

[bib27] Lieberman-Aiden E., van Berkum N.L., Williams L., Imakaev M., Ragoczy T., Telling A., Amit I., Lajoie B.R., Sabo P.J., Dorschner M.O. (2009). Comprehensive mapping of long-range interactions reveals folding principles of the human genome. Science.

[bib28] Lu L., Liu X., Huang W.K., Giusti-Rodríguez P., Cui J., Zhang S., Xu W., Wen Z., Ma S., Rosen J.D. (2020). Robust hi-C maps of enhancer-promoter interactions reveal the function of non-coding genome in neural development and diseases. Mol. Cell.

[bib29] Lua R., Borovinskiy A.L., Grosberg A.Y. (2004). Fractal and statistical properties of large compact polymers: a computational study. Polymer.

[bib30] Mashaghi A., Van Wijk R.J., Tans S.J. (2014). Circuit topology of proteins and nucleic acids. Structure.

[bib31] Mirny L.A. (2011). The fractal globule as a model of chromatin architecture in the cell. Chromosom. Res..

[bib32] Mugler A., Tans S.J., Mashaghi A. (2014). Circuit topology of self-interacting chains: implications for folding and unfolding dynamics. Phys. Chem. Chem. Phys..

[bib33] Nagano T., Lubling Y., Stevens T.J., Schoenfelder S., Yaffe E., Dean W., Laue E.D., Tanay A., Fraser P. (2013). Single-cell Hi-C reveals cell-to-cell variability in chromosome structure. Nature.

[bib34] Nagano T., Lubling Y., Várnai C., Dudley C., Leung W., Baran Y., Mendelson Cohen N., Wingett S., Fraser P., Tanay A. (2017). Cell-cycle dynamics of chromosomal organization at single-cell resolution. Nature.

[bib35] Nasmyth K. (2001). Disseminating the genome: joining, resolving, and separating sister chromatids during mitosis and meiosis. Annu. Rev. Genet..

[bib36] Norton H.K., Emerson D.J., Huang H., Kim J., Titus K.R., Gu S., Bassett D.S., Phillips-cremins J.E. (2018). Detecting hierarchical genome folding with network modularity. Nat. Methods.

[bib37] Ong C.-T., Corces V.G. (2014). CTCF: an architectural protein bridging genome topology and function. Nat. Rev. Genet..

[bib38] Orlandini E., Marenduzzo D., Michieletto D. (2019). Synergy of topoisomerase and structural-maintenance-of-chromosomes proteins creates a universal pathway to simplify genome topology. Proc. Natl. Acad. Sci. U S A.

[bib39] Pouokam M., Cruz B., Burgess S., Segal M.R., Vazquez M., Arsuaga J. (2019). The Rabl configuration limits topological entanglement of chromosomes in budding yeast. Sci. Rep..

[bib40] Pucéat M. (2021). Capturing chromosome conformation. Methods Mol. Biol..

[bib41] Rao S.S.P., Huntley M.H., Durand N.C., Stamenova E.K., Bochkov I.D., Robinson J.T., Sanborn A.L., Machol I., Omer A.D., Lander E.S., Aiden E.L. (2014). A 3D map of the human genome at kilobase resolution reveals principles of chromatin looping. Cell.

[bib42] Rosa A., Everaers R. (2008). Structure and dynamics of interphase chromosomes. PLoS Comput. Biol..

[bib43] Rougier N.P. (2016). https://gist.github.com/rougier/e5eafc276a4e54f516ed5559df4242c0.

[bib44] Rowley M.J., Corces V.G. (2018). Organizational principles of 3D genome architecture. Nat. Rev. Genet..

[bib45] Sanborn A.L., Rao S.S.P., Huang S.C., Durand N.C., Huntley M.H., Jewett A.I., Bochkov I.D., Chinnappan D., Cutkosky A., Li J. (2015). Chromatin extrusion explains key features of loop and domain formation in wild-type and engineered genomes. Proc. Natl. Acad. Sci. U S A.

[bib46] Satarifard V., Heidari M., Mashaghi S., Tans S.J., Ejtehadi M.R., Mashaghi A. (2017). Topology of polymer chains under nanoscale confinement. Nanoscale.

[bib47] Sazer S., Schiessel H. (2018). The biology and polymer physics underlying large-scale chromosome organization. Traffic.

[bib48] Scalvini B., Sheikhhassani V., Mashaghi A. (2021). Topological principles of protein folding. Phys. Chem. Chem. Phys..

[bib49] Scalvini B., Sheikhhassani V., Woodard J., Aupič J., Dame R.T., Jerala R., Mashaghi A. (2020). Topology of folded molecular chains: from single biomolecules to engineered origami. Trends Chem..

[bib50] Schullian O., Woodard J., Tirandaz A., Mashaghi A. (2020). A circuit topology approach to categorizing changes in biomolecular structure. Front. Phys..

[bib51] Serizay J., Ahringer J. (2018). ScienceDirect genome organization at different scales : nature , formation and function. Curr. Opin. Cell Biol..

[bib52] Spielmann M., Lupiáñez D.G., Mundlos S. (2018). Structural variation in the 3D genome. Nat. Rev. Genet..

[bib53] Stephens A.D., Banigan E.J., Marko J.F. (2019). Chromatin’s physical properties shape the nucleus and its functions. Curr. Opin. Cell Biol..

[bib54] Stevens T.J., Lando D., Basu S., Atkinson L.P., Cao Y., Lee S.F., Leeb M., Wohlfahrt K.J., Boucher W., O’Shaughnessy-Kirwan A. (2017). 3D structures of individual mammalian genomes studied by single-cell Hi-C. Nature.

[bib55] Virtanen P., Gommers R., Oliphant T.E., Haberland M., Reddy T., Cournapeau D., Burovski E., Peterson P., Weckesser W., Bright J. (2020). SciPy 1.0: fundamental algorithms for scientific computing in python. Nat. Methods.

[bib56] Watts D.J., Strogatz S.H. (1998). Strogatz - small world network nature. Nature.

