# Supplementary material for: Circuit topology analysis of cellular genome reveals signature motifs, conformational heterogeneity, and scaling
Source: iScience. 2022 Feb 5;25(3):103866. doi: 10.1016/j.isci.2022.103866 (PMC8861635; doi:10.1016/j.isci.2022.103866)
Supplement: Document S1. Figures S1–S9 and Tables S1–S6 [file mmc1.pdf]

iScience, Volume *25*

Supplemental information

Circuit topology analysis of cellular genome reveals signature motifs, conformational heterogeneity, and scaling

Barbara Scalvini, Helmut Schiessel, Anatoly Golovnev, and Alireza Mashaghi

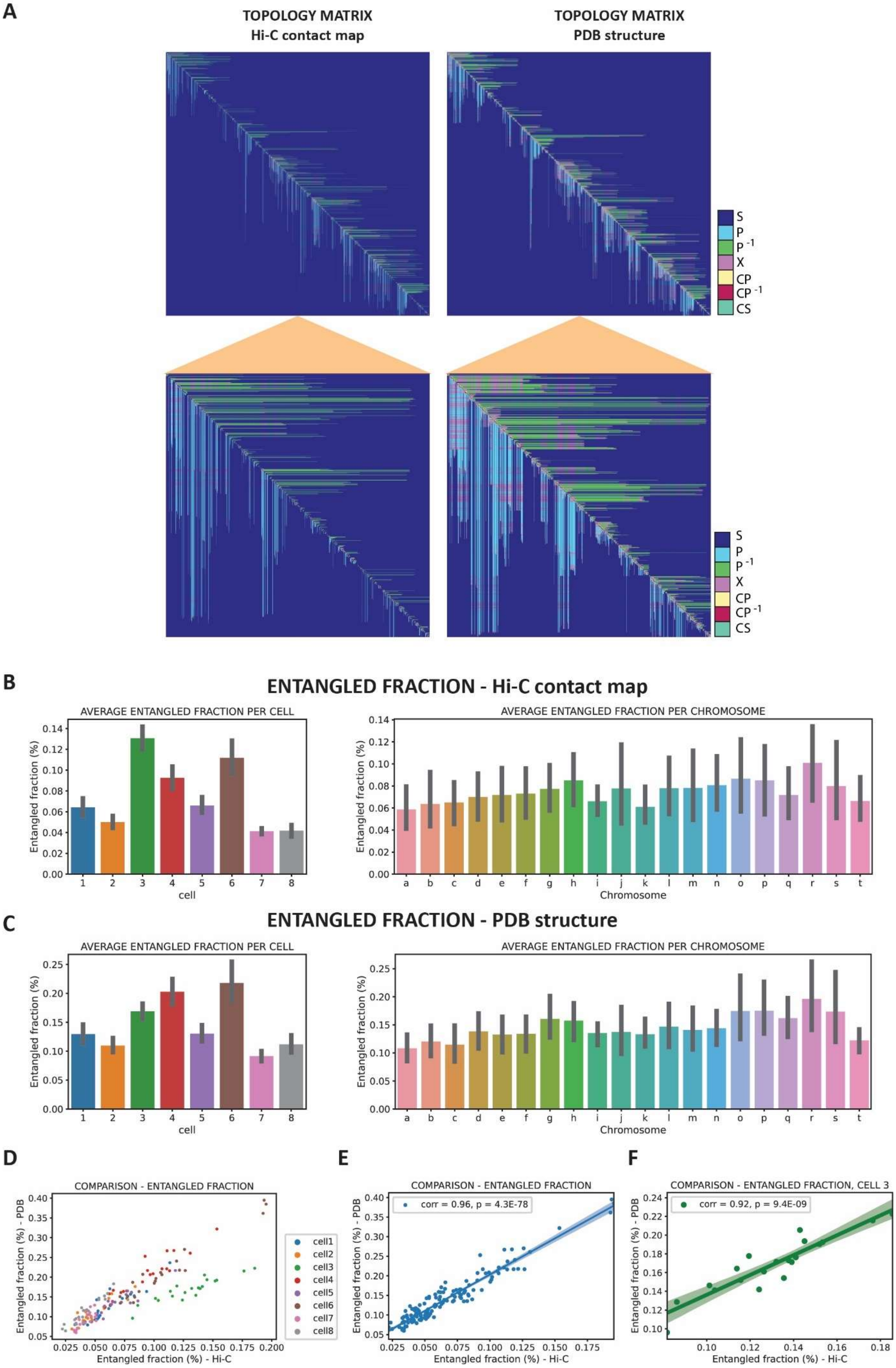
A
TOPOLOGY MATRIX
Hi-C contact map
TOPOLOGY MATRIX
PDB structure
S
P
P -1
X
CP
CP -1
CS
B
ENTANGLED FRACTION - Hi-C contact map
AVERAGE ENTANGLED FRACTION PER CELL
Entangled fraction (%)
cell
AVERAGE ENTANGLED FRACTION PER CHROMOSOME
Chromosome
C
ENTANGLED FRACTION - PDB structure
AVERAGE ENTANGLED FRACTION PER CELL
AVERAGE ENTANGLED FRACTION PER CHROMOSOME
D
COMPARISON - ENTANGLED FRACTION
Entangled fraction (%) - PDB
Entangled fraction (%) - Hi-C
cell1
cell2
cell3
cell4
cell5
cell6
cell7
cell8
E
COMPARISON - ENTANGLED FRACTION
corr = 0.96, p = 4.3E-78
F
COMPARISON - ENTANGLED FRACTION, CELL 3
corr = 0.92, p = 9.4E-09

Figure S1. Topology matrices can be retrieved directly from Hi-C contact maps. Related to Figure 1 and 2. The only requirement needed to create a topology matrix is a list of the contact indexes which characterize the folded chain. Therefore, topology matrices can be derived directly from Hi-C maps of chromosomes. Examples on how to generate CT matrices from Hi-C data can be found in the Jupyter notebook provided with the paper. **A** The figure shows a comparison between the topology matrix of chromosome 1, cell 1 derived from 3D structure and from its Hi-C contact list. The topology matrix retrieved from 3D structure was calculated with spatial cutoff = 1.75, as this choice provided a similar number of contacts as those reported in the Hi-C map (7579 for Hi-C map, 7311 for the 3D structure), and therefore, a similar resolution between the two topology matrices. The characteristic topological features associated to the chromosome structure are clearly identifiable in both topology matrices. However, patterns in the topology matrix from the 3D structure appear thicker. This discrepancy is due to the different procedure for contact identification allowed by the two methods. The 3D structures allow for a spatial understanding of contacts and contact sites. Often neighboring contact sites will participate in the same contacts: for example, if contact sites (*a*, *b*) participate in a contact, it is very likely that also neighboring contact sites *a-1* and *a+1* participate in a contact with contact site *b* (they are found to be at a distance which is shorter than the chosen cutoff). Contacts (*a-1*, *b*) and (*a+1*, *b*) will most likely have the same topological relation as (*a*, *b*). Thus, the pattern in the topology matrix will be thicker than it would be if we only considered contact (*a*, *b*). Moreover, considering neighboring contact sites leads to an increased detection of concerted relationships (CP, CS) along the diagonal, as we can see from the matrix detail in the Figure. The overall results of the analysis are not affected by these differences. However, we decided to perform our analysis on 3D model structures, as that allowed us to study the system with varying degree of spatial resolution. Moreover, the higher definition of topological patterns can be beneficial when identifying characteristic features in the matrix. The analysis pipeline showed in this paper can be generalized also to ensemble Hi-C data, once a threshold for contact likelihood is set for contact filtering. **B** Bar plot of the entangled fraction per cell (averaged over chromosomes) and per chromosome (averaged over cells), calculated from single cell Hi-C contacts maps. Error bars show a 95% confidence interval for the mean. **C** Bar plot of the entangled fraction per cell (averaged over chromosomes) and per chromosome (averaged over cells), calculated from 3D structures. Error bars show a 95% confidence interval for the mean. **D** Scatter plot of the entangled fraction calculated starting from 3D structures versus the entangled fraction calculated starting from single cell Hi-C maps. Each dot represents a chromosome. Colors represent the cell to which the chromosomes belong. Cell 3 deviates from the linear relationship identified for the other cells. **E** Linear regression of the entangled fraction calculated starting from 3D structures versus the entangled fraction calculated starting from single cell Hi-C maps, excluding cell 3. The label reports the Pearson correlation coefficient and its p value. **F** Linear regression of the entangled fraction calculated starting from 3D structures versus the entangled fraction calculated starting from single cell Hi-C maps, for cell 3. The label reports the Pearson correlation coefficient and its p value.

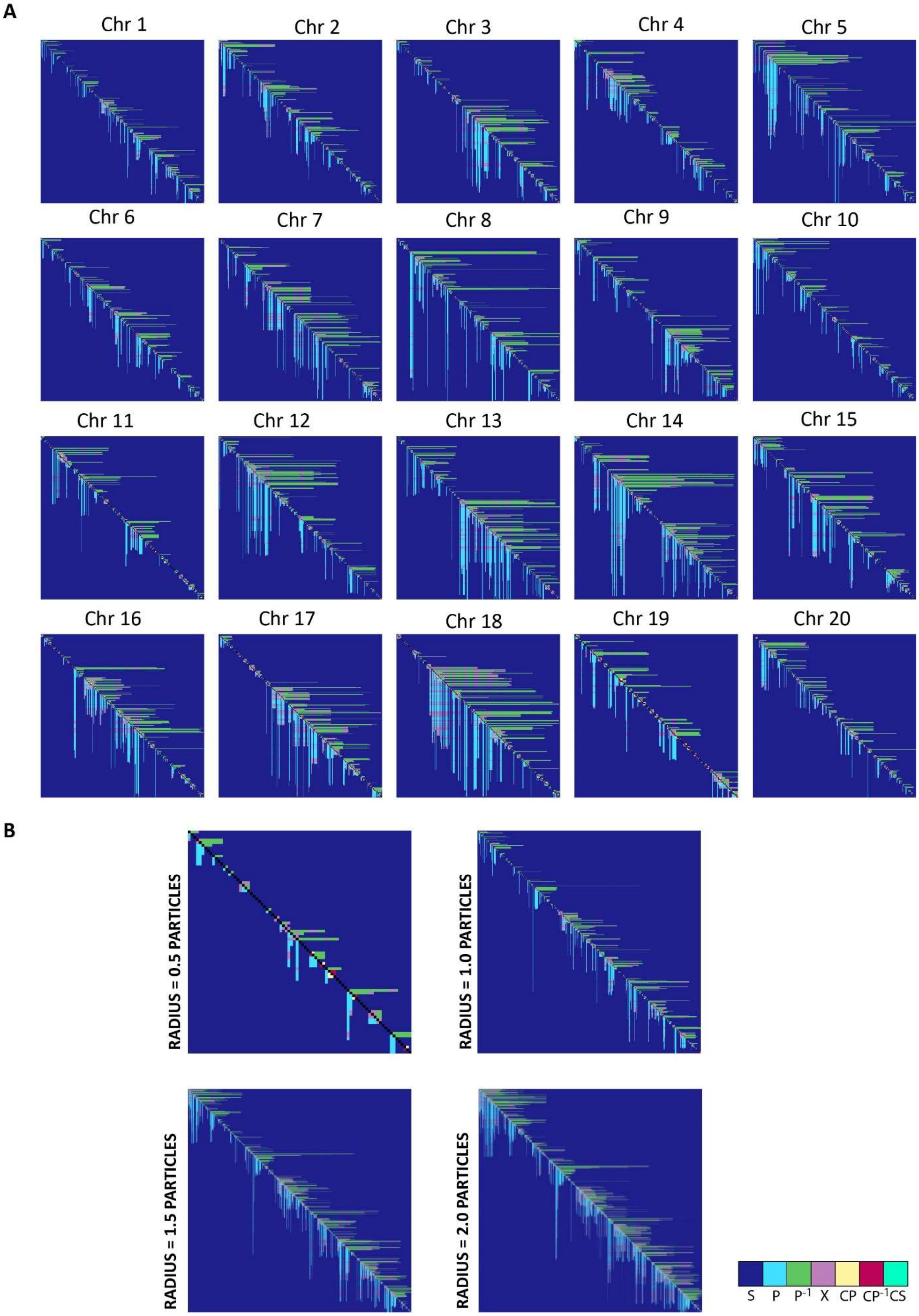
A
Chr 1
Chr 2
Chr 3
Chr 4
Chr 5
Chr 6
Chr 7
Chr 8
Chr 9
Chr 10
Chr 11
Chr 12
Chr 13
Chr 14
Chr 15
Chr 16
Chr 17
Chr 18
Chr 19
Chr 20
B
RADIUS = 0.5 PARTICLES
RADIUS = 1.0 PARTICLES
RADIUS = 1.5 PARTICLES
RADIUS = 2.0 PARTICLES
S P P-1 X CP CP-1 CS

Figure S2. Topology matrices of all chromosomes for cell 1. Related to Figure 2. A All matrices were calculated with a cutoff of 1 particle. First neighbors were excluded from contact detection. Rows and columns represent contact indexes. Indexes are computed left end to right end along the chain. **B** Topology matrix of chromosome 1, cell 1, calculated with different cutoff radii. L-pattern structures are clearly identifiable for all cutoff choices. For calculations with radius r_c = 2.0 we recommend excluding first and second neighbors: second-neighbors inclusion might cause artifacts along the diagonal of the matrix.

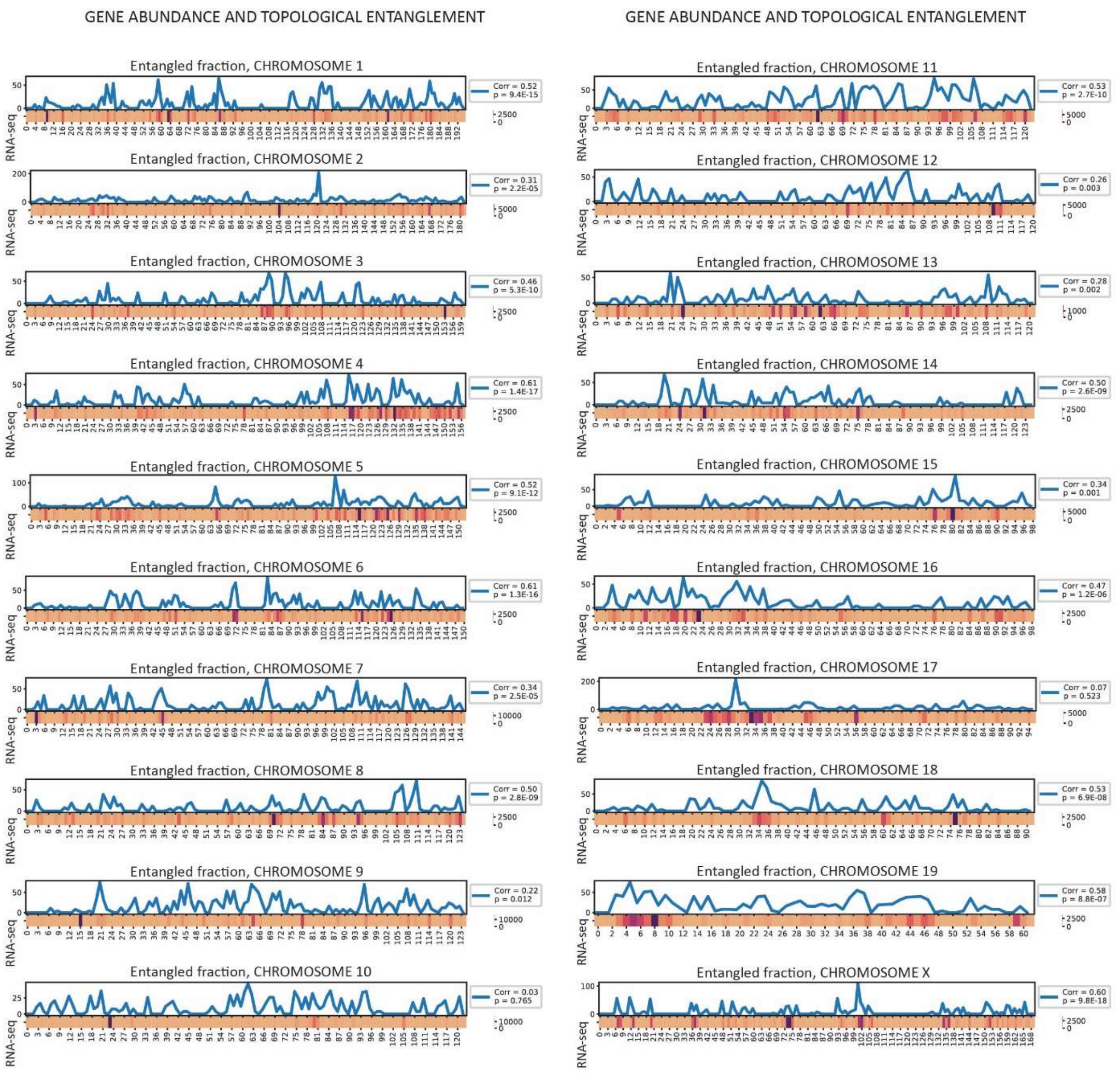

Figure S3. The topological information can be projected onto chromosomal structure and coupled with relevant biological parameters. Related to Figure 3. Plot of the entanglement fraction trace calculated over population Hi-C maps, coupled with a heatmap of gene abundance data as retrieved by nuclear RNA-seq, for all chromosomes. The data was coarse-grained in 1Mb bins. Gene abundance lower than 50 for Mb was set to zero, and a threshold of 250 counts was set over population Hi-C contacts, in order to increase the signal to noise ratio. The label shows the correlation between entanglement fraction and gene abundance. All chromosomes display statistically significant positive correlation between the two parameters, except for chromosome 10 and 17.

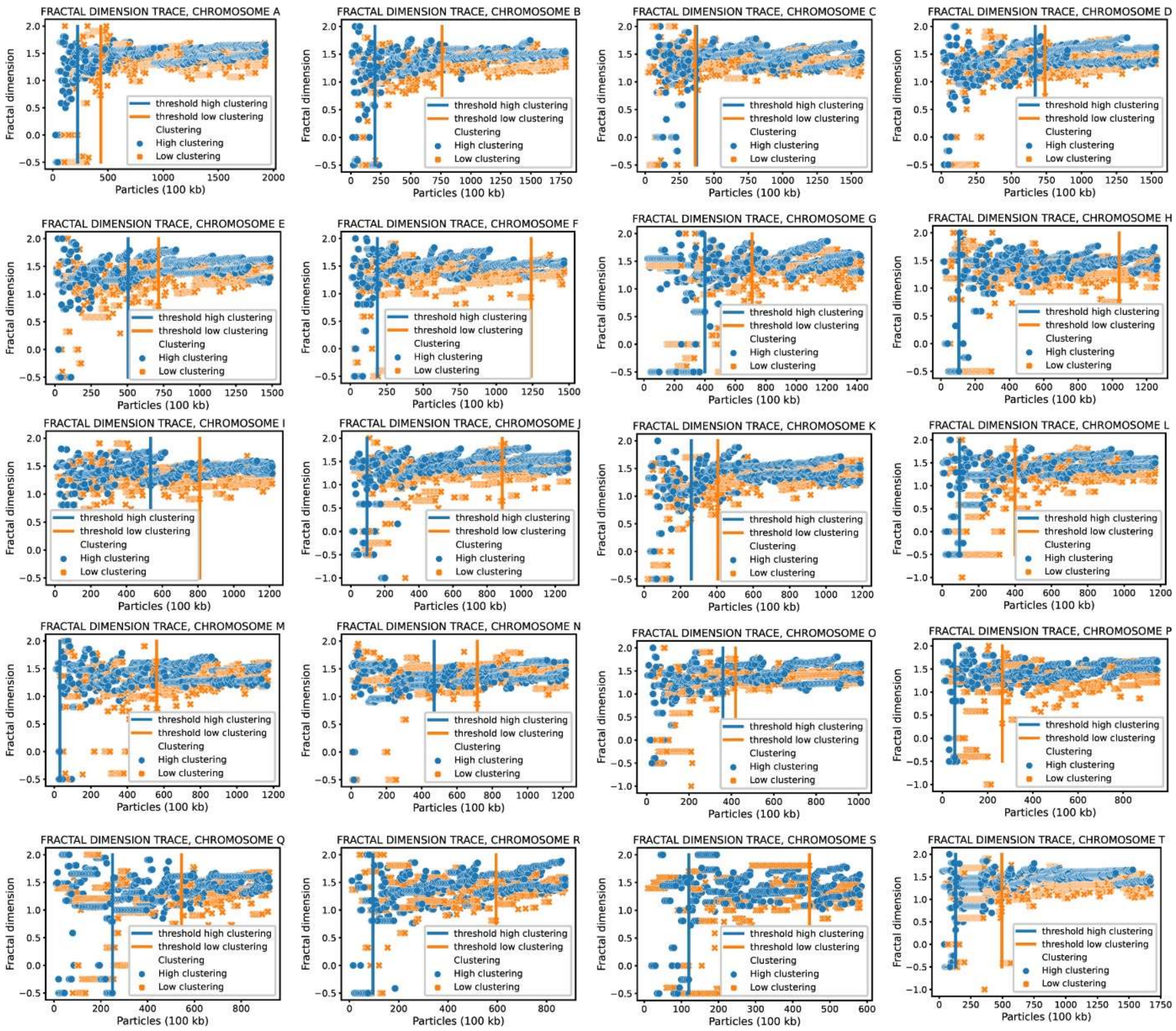

Figure S4. Length scale and fractal dimension. Related to Figure 7. Trace analysis for fractal dimension, for each chromosome, all cells. The vertical bars indicate the convergence threshold for the traces. The high clustering cell group always reaches convergence for a smaller number of particles than the low clustering cell group, with the only exception of chromosome C.

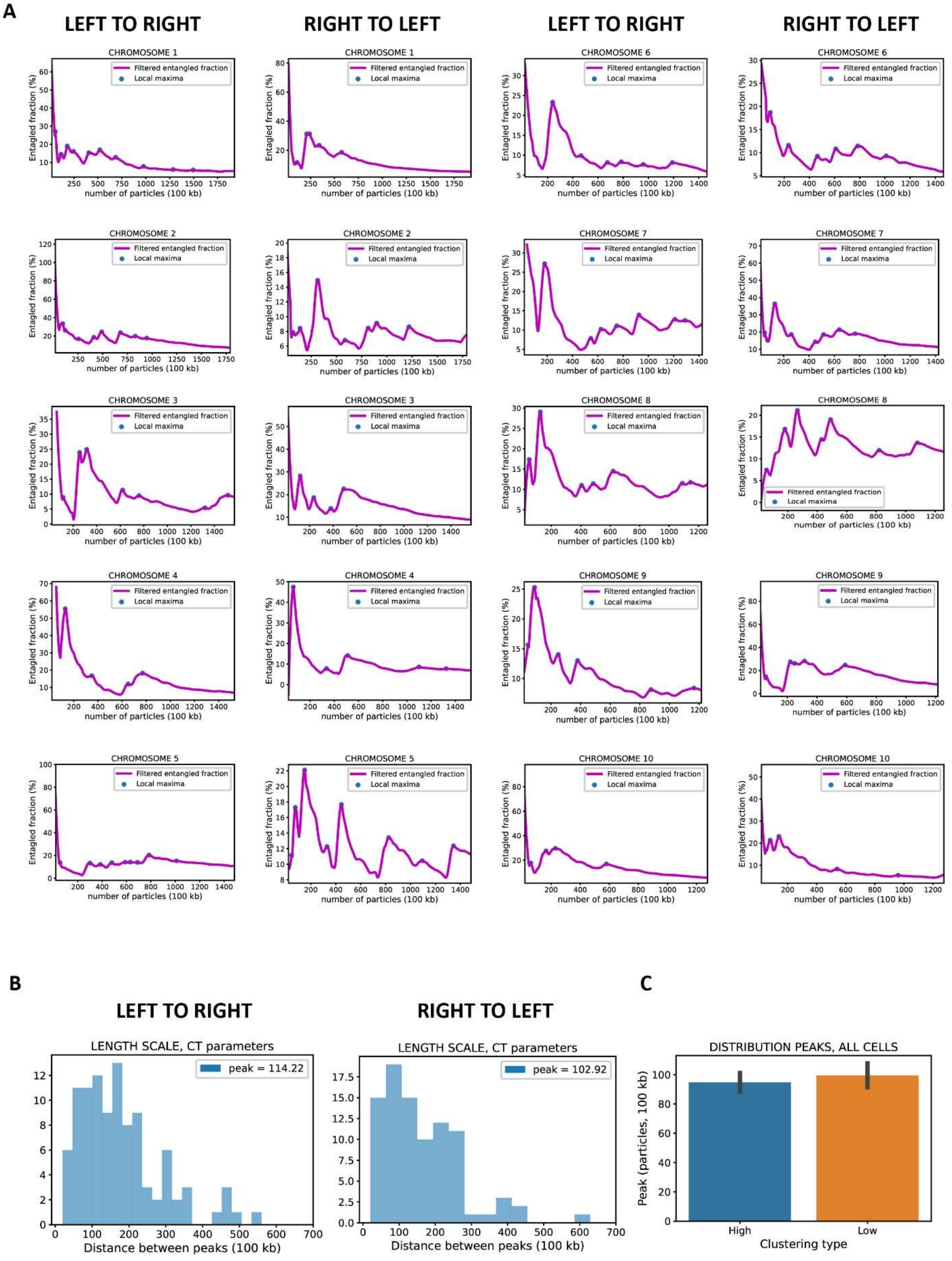

Figure S5. Length scale analysis from left end to right and of the chain and vice versa. Related to Figure 7. A Comparison of the cumulative traces of the entangled fraction of chromosome 1 to 10, cell 1, obtained by processing the chain left end to right end and vice versa. **B** Distribution of distances between local maxima in the trace of entangled fraction, for

cell 1, obtained by processing the chain left end to right end and vice versa. **C** Bar plot of peaks of the distributions of distance between local maxima in entangled traces for all cells, obtained by processing the chain from right end to left end. Error bars show a 95% confidence interval for the mean.

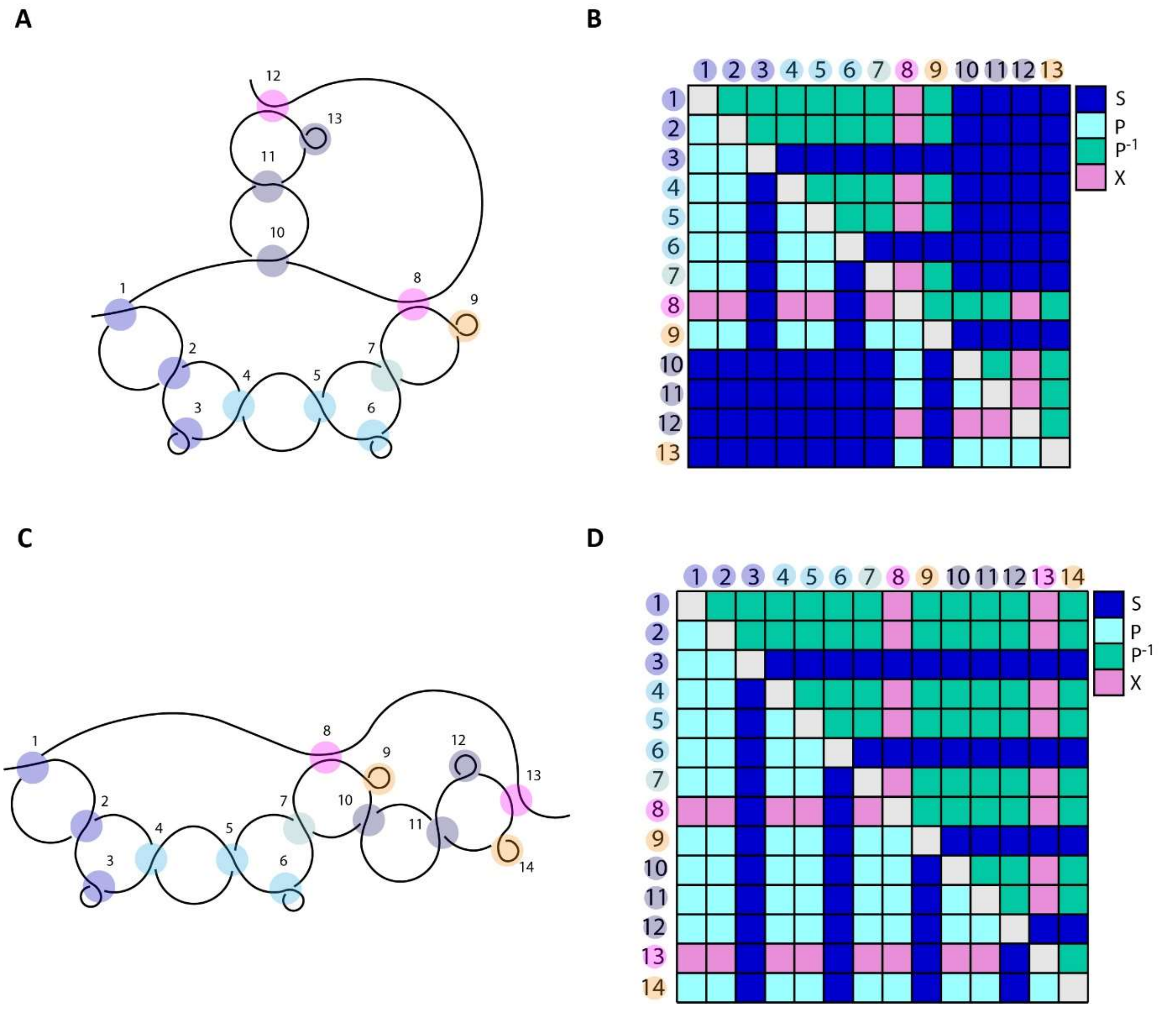

Figure S6. Variations of the L-loop model. Related to Figure 8. A L-loop arrangement leading to overlapping L-patterns in the topology matrix. **B** Topology matrix presenting overlapping L-patterns, corresponding to the contact arrangement in A. **C** L-loop arrangement leading to L-patterns with multiple (two) cross stripes in the topology matrix. **D** Topology matrix presenting L-patterns with multiple (two) cross stripes, corresponding to the contact arrangement in C.

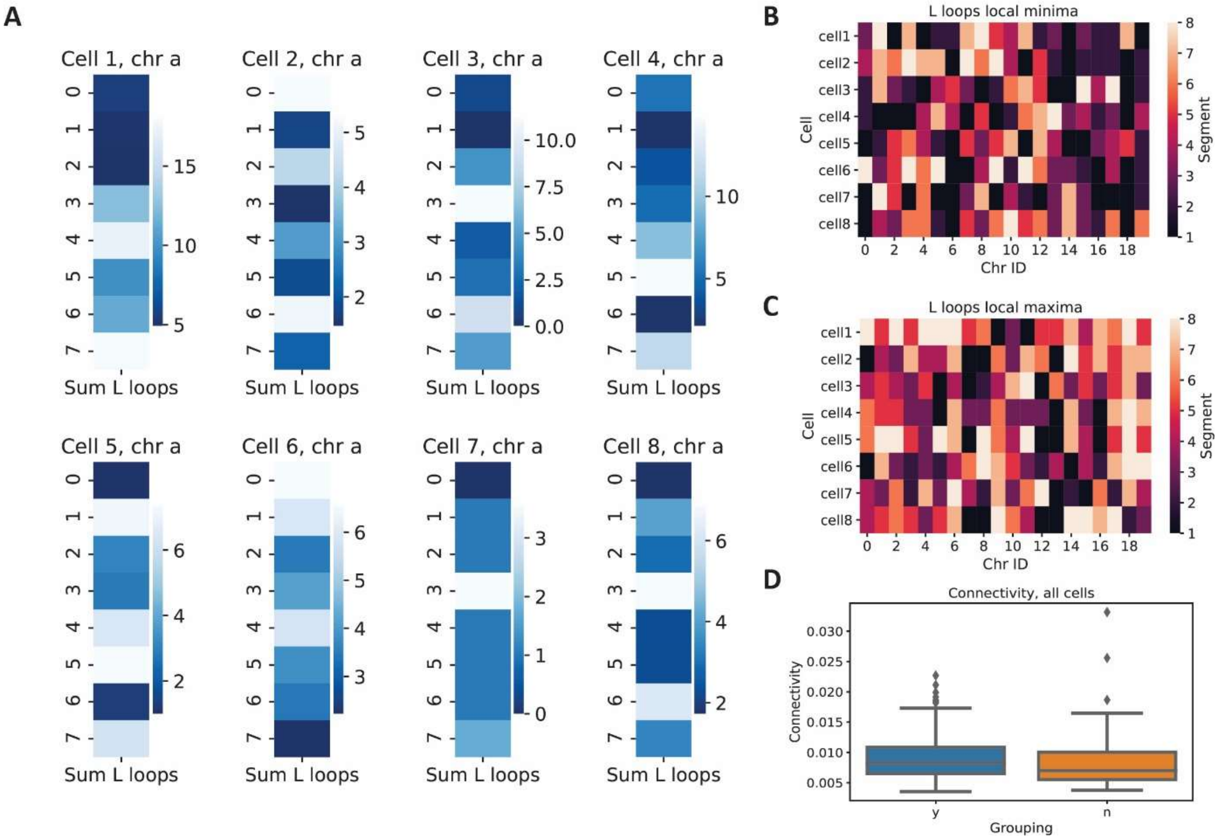

Figure S7. The local distribution of L-loops can inform us about the overall topological properties of the chromosomes. Related to Figure 9. A Chromosome 'bar codes' for chromosome a, all cells: the color of the heatmap indicates the sum of contacts stored in L-loops in each chromosome segment. The chromosomes were divided into 8 segments. **B** Heatmap indicating in which segment of each chromosome the minimum number of L-loops is reached. **C** Heatmap indicating in which segment of each chromosome the maximum number of L-loops is reached. **D** By observing map B and C, a prevalence of black color (Segment 1) can be found in map B with respect to C. Such a trend would indicate that chromosomes have more often minima rather than maxima in their first segment. Therefore, we created a grouping based on whether a chromosome reaches maxima in its first segment in at least one of the 8 cells. Chromosomes that do reach maxima in their first segment (grouping 'y') seem to have slightly higher connectivity on average than other chromosomes ($p = 0.048$, calculated by Mann-Whitney U non parametric test). Whiskers in the boxplot are extended to 1.5 IQR.

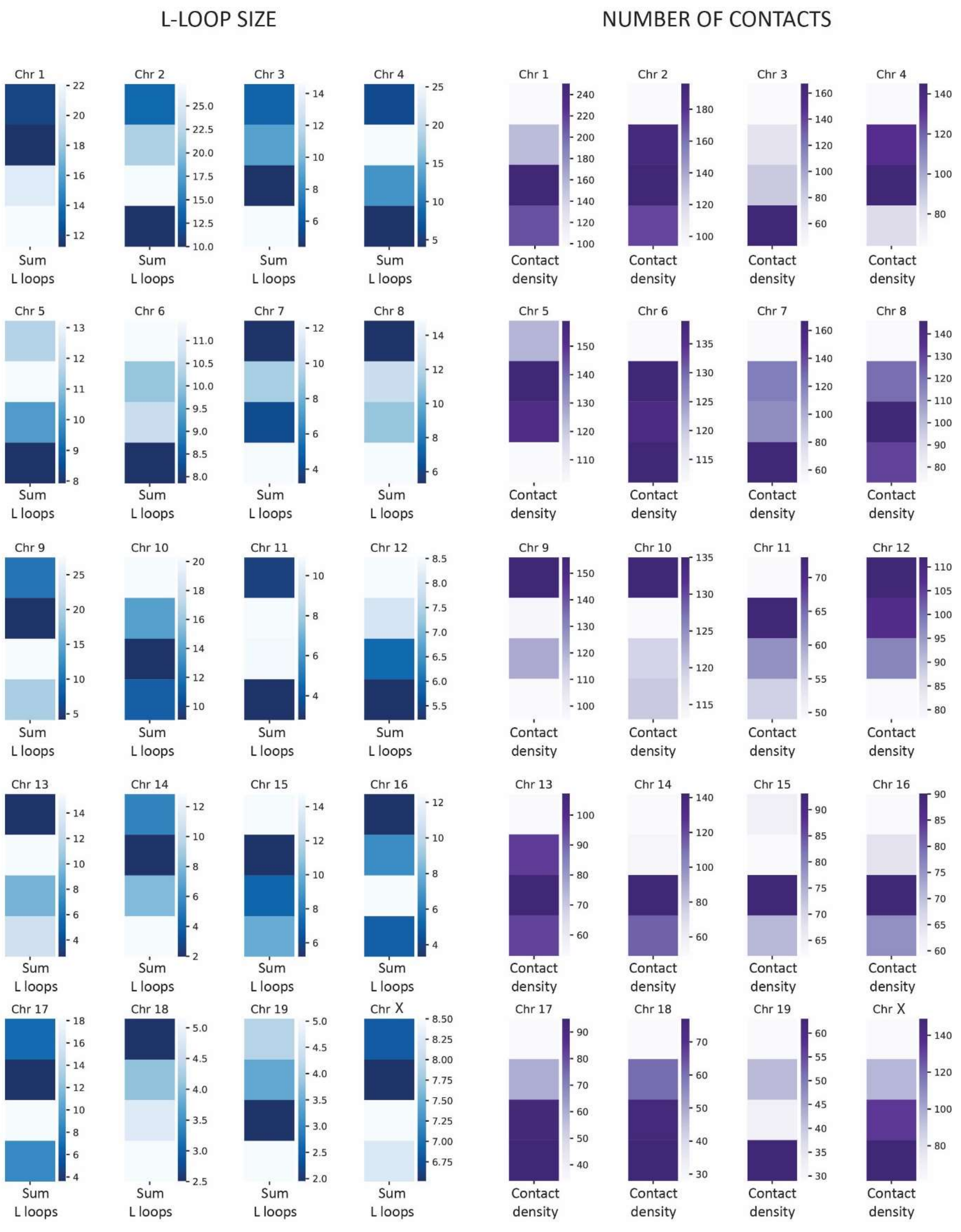

Figure S8. Local analysis of L-loops and number of contacts. Related to Figure 9. Heatmaps of the sum of contacts enveloped by L-loops (in blue) and number of contacts (in purple), for chromosomes divided into 4 segments. The figure shows the heatmap for all chromosomes in cell 1.

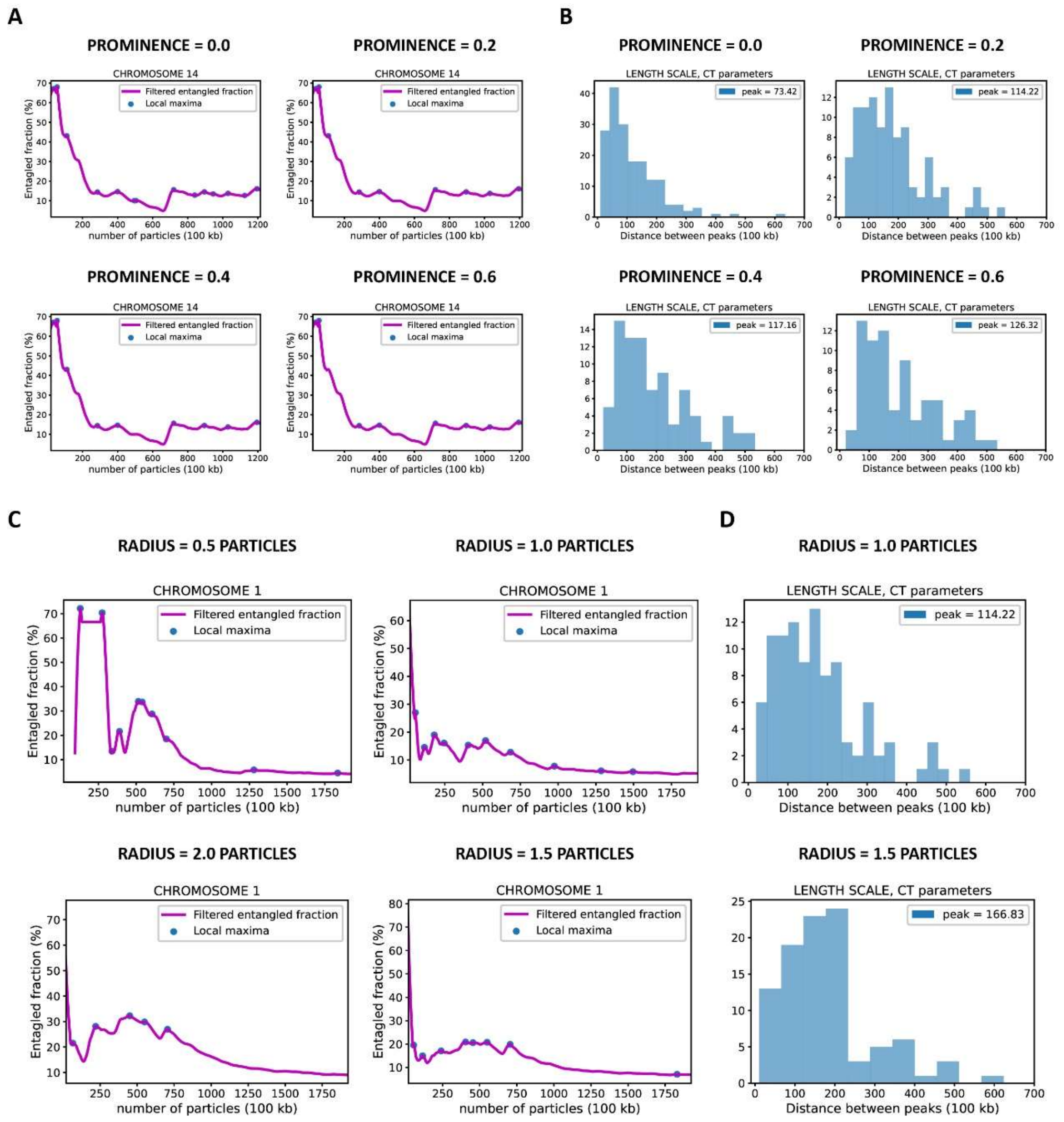

Figure S9. Length scale analysis for different values of maxima prominence and cutoff radii. Related to Figure 7. A Cumulative trace of entangled fraction of chromosome 1, cell 1, and maxima detection (scatter dots) for prominence= 0.0, 0.2, 0.4, 0.6. Prominence 0.2 and 0.4 yield the same maxima positions. **B** Distribution of distances between local maxima in the trace of entangled fraction, for cell one, obtained by detecting maxima with different values of prominence. **C** Cumulative trace of the entangled fraction for chromosome 1, cell 1 for different cutoff values. Dots represent local maxima in the curve. **D** Distribution of distances between local maxima in the trace of entangled fraction, for cell one, obtained for two different cutoffs. Radii 1.0 and 1.5 particles were deemed the most reliable after visual inspection of the contacts in the chain.

Chromosome	Correlation	Pvalue
**chr1**	0.51	1.2e-14
**chr2**	0.31	2.4e-05
**chr3**	0.46	5.9e-10
**chr4**	0.61	1.7e-17
**chr5**	0.51	9.8e-12
**chr6**	0.61	1.3e-16
**chr7**	0.34	2.7e-05
**chr8**	0.5	3,00E-09
**chr9**	0.22	0.013
**chr10**	0.03	0.774
**chr11**	0.53	2.9e-10
**chr12**	0.26	0.004
**chr13**	0.28	0.002
**chr14**	0.5	3,00E-09
**chr15**	0.34	0.001
**chr16**	0.46	1.2e-06
**chr17**	0.07	0.525
**chr18**	0.52	8.1e-08
**chr19**	0.58	9,00E-07
**chrX**	0.59	1.4e-17

Table S1. Entangled relation from Hi-C population map correlates locally with gene expression: no threshold for abundance levels. Related to Table 1. Correlation coefficient and p values for entanglement fraction and gene abundance data as measured by nuclear RNA-seq. The data used to obtain these correlation coefficients were coarse-grained in 1Mb bins. A threshold of 250 counts was set over population Hi-C contacts.

Chromosome	Correlation	Pvalue
**chr1**	0.4	7.4e-09
**chr2**	0.3	3,00E-05
**chr3**	0.42	4.4e-08
**chr4**	0.42	2.7e-08
**chr5**	0.39	5.7e-07
**chr6**	0.41	1.4e-07
**chr7**	0.19	0.02
**chr8**	0.36	4.4e-05
**chr9**	0.16	0.083
**chr10**	0.01	0.949
**chr11**	0.42	1,00E-06
**chr12**	0.24	0.007
**chr13**	0.19	0.037
**chr14**	0.47	3,00E-08
**chr15**	0.2	0.045
**chr16**	0.39	7.4e-05
**chr17**	-0.03	0.775
**chr18**	0.64	6.9e-12
**chr19**	0.23	0.078
**chrX**	0.48	2.2e-11

Table S2. Entangled relation from Hi-C population map correlates locally with gene expression: low Hi-C counts threshold set to 100. Related to Table 1. Correlation coefficient and p values for entanglement fraction and gene abundance data as measured by nuclear RNA-seq. The data used to obtain these correlation coefficients were coarse-grained in 1Mb bins. Gene abundance lower than 50 for Mb was set to zero, and a threshold of 100 counts was set over population Hi-C contacts.

Chromosome	Correlation	Pvalue
**chr1**	0.41	2.1e-09
**chr2**	0.34	2.2e-06
**chr3**	0.42	4.4e-08
**chr4**	0.55	3.9e-14
**chr5**	0.51	2.8e-11
**chr6**	0.5	8.5e-11
**chr7**	0.29	0.0004
**chr8**	0.49	6.3e-09
**chr9**	0.21	0.018
**chr10**	0.05	0.599
**chr11**	0.52	1.1e-09
**chr12**	0.29	0.001
**chr13**	0.24	0.007
**chr14**	0.47	2,00E-08
**chr15**	0.3	0.003
**chr16**	0.37	0.00019
**chr17**	0.02	0.826
**chr18**	0.49	7.5e-07
**chr19**	0.49	5.2e-05
**chrX**	0.57	6.7e-16

Table S3. Entangled relation from Hi-C population map correlates locally with gene expression: low Hi-C counts threshold set to 200. Related to Table 1. Correlation coefficient and p values for entanglement fraction and gene abundance data as measured by nuclear RNA-seq. The data used to obtain these correlation coefficients were coarse-grained in 1Mb bins. Gene abundance lower than 50 for Mb was set to zero, and a threshold of 200 counts was set over population Hi-C contacts.

Chromosome	Correlation	Pvalue
**chr1**	0.51	3.4e-14
**chr2**	0.26	0.00029
**chr3**	0.4	1.9e-07
**chr4**	0.67	4.2e-22
**chr5**	0.41	1.7e-07
**chr6**	0.55	4.5e-13
**chr7**	0.33	5.4e-05
**chr8**	0.41	2.7e-06
**chr9**	0.22	0.012
**chr10**	0.04	0.639
**chr11**	0.55	2.8e-11
**chr12**	0.28	0.002
**chr13**	0.21	0.02
**chr14**	0.5	2.1e-09
**chr15**	0.29	0.004
**chr16**	0.38	9.5e-05
**chr17**	0.07	0.514
**chr18**	0.44	1.2e-05
**chr19**	0.73	1.2e-11
**chrX**	0.6	6.7e-18

Table S4. Entangled relation from Hi-C population map correlates locally with gene expression: low Hi-C counts threshold set to 300. Related to Table 1. Correlation coefficient and p values for entanglement fraction and gene abundance data as measured by nuclear RNA-seq. The data used to obtain these correlation coefficients were coarse-grained in 1Mb bins. Gene abundance lower than 50 for Mb was set to zero, and a threshold of 300 counts was set over population Hi-C contacts.

Shapiro High	Shapiro Random	Levene High-Random	Kolmogorov High-Random
8.06e-08	3.98e-08	6.61e-08	1.45e-41
8.06e-08	0.00012	1.88e-09	5.71e-40
8.06e-08	1.08e-08	2.05e-07	1.45e-41
8.06e-08	0.00092	8.99e-14	1.45e-41
8.06e-08	3.84e-06	4.13e-08	1.45e-41
8.06e-08	4.36e-07	5.62e-09	1.45e-41
8.06e-08	4.04e-09	1.35e-07	1.45e-41
8.06e-08	3.15e-08	1.01e-07	2.76e-43
8.06e-08	7.60e-08	1.14e-07	1.45e-41
8.06e-08	2.35e-13	5.37e-05	2.76e-43

Table S5. P values of statistical tests performed on the fractal dimension distribution of 10 extractions of 80 random topology matrices, in comparison with fractal dimension distribution of chromosomes from the high clustering group. Related to Figure 6. Shapiro test was used to test normality of the fractal dimension distribution. Levene's Test was used to test the homogeneity of variance between random distribution and high clustering distribution. The Kolmogorov-Smirnov test was used to compare the two distributions for each random extraction.

Shapiro Low	Shapiro Random	Levene Low-Random	Kolmogorov Low-Random
2.14e-05	3.98e-08	1.75e-08	3.48e-45
2.14e-05	0.00012	2.11e-10	3.48e-45
2.14e-05	1.08e-08	5.09e-08	2.17e-47
2.14e-05	0.00092	5.71e-15	3.48e-45
2.14e-05	3.84e-06	7.23e-09	2.17e-47
2.14e-05	4.36e-07	9.94e-10	3.48e-45
2.14e-05	4.04e-09	3.72e-08	2.17e-47
2.14e-05	3.15e-08	2.63e-08	2.17e-47
2.14e-05	7.60e-08	3.048e-08	2.17e-47
2.14e-05	2.35e-13	2.80e-05	2.17e-47

Table S6. P values of statistical tests performed on the fractal dimension distribution of 10 extractions of 80 random topology matrices, in comparison with fractal dimension distribution of chromosomes from the low clustering group. Related to Figure 6. Shapiro test was used to test normality of the fractal dimension distribution. Levene's Test was used to test the homogeneity of variance between random distribution and low clustering distribution. The Kolmogorov-Smirnov test was used to compare the two distributions for each random extraction.
